# Epileptic seizure detection from electroencephalogram signals based on 1D CNN-LSTM deep learning model using discrete wavelet transform

**DOI:** 10.1038/s41598-025-18479-9

**Published:** 2025-09-25

**Authors:** Homa Kashefi Amiri, Masoud Zarei, Mohammad Reza Daliri

**Affiliations:** 1 Department of Bioengineering, University of Pittsburgh, 3700 O’Hara St, Pittsburgh, PA 15260 USA; 2 Biomedical Engineering Department, School of Electrical Engineering, Iran University of Science and Technology (IUST), Narmak, Tehran, 16846-13114 Iran

**Keywords:** Convolutional neural network, Long-short term memory, Electroencephalogram, Epileptic seizure detection, Discrete wavelet transform, Biomedical engineering, Diseases

## Abstract

Excessive electrical activity in the brain causes epileptic seizures which can be detected through Electroencephalogram (EEG) signals. The research aims to identify epileptic seizures using EEG records automatically. Firstly, EEG bands are extracted using Discrete Wavelet Transform (DWT) and concatenated. Secondly, the resulting feature vector is fed into a 1-dimensional Convolutional Neural Network (CNN) to extract spatial information. The Long-Short Term Memory (LSTM) layer then receives the feature maps in order to extract the temporal information. Ultimately, a fully connected layer will use the generated spatiotemporal features as input to categorize the signal. Results show that the suggested model performs well on the following datasets: the TUSZ corpus, which has 94.32% accuracy, 86.08% Kappa value, and 79.01% GDR; the BONN dataset, which has 97.24% accuracy, 97.92% Kappa value, and 99.18% GDR; and the CHB-MIT dataset, which has 96.94% accuracy, 94.33% Kappa value, and 96.36% GDR. The computational complexity for BONN, CHB-MIT, and TUSZ datasets are $\:3.07\times\:{10}^{7}$, $\:1.67\times\:{10}^{6}$ and $\:1.67\times\:{10}^{6}$ respectively. The performance of several popular machine learning classifiers is compared with the proposed model. The results show that the model outperforms existing approaches. The model’s strong performance is largely due to the CNN’s ability to effectively extract meaningful spatial features.

**Supplementary Information:**

The online version contains supplementary material available at 10.1038/s41598-025-18479-9.

## Introduction

Epilepsy is a life-threatening brain dysfunction which causes seizures^1^. The World Health Organization (WHO) reports that epilepsy affects over 50 million people worldwide^2^. There are causes for epilepsy such as skull fractures, brain tumors, and other conditions contributing to its onset^3^. Early detection and timely intervention are critical in clinical settings. As a result, developing an automated system for detecting epileptic seizures is essential for improving patient care systems. Magnetic resonance imaging (MRI), functional magnetic resonance imaging (fMRI), electroencephalography (EEG), and computed tomography (CT) scans are among the methods used to investigate and discover epilepsy^4^. The detection and recognition of epileptic seizures are often done by EEG signals^5^. In patients diagnosed with epilepsy, EEG activity is divided into two phases: the interictal and ictal phases^6^; the ictal phase is the outbreak period of epilepsy, and the interictal phase is the period between seizures. In addition to the time-consuming nature of diagnosing epileptic seizures from the EEG signal, the misdiagnosis led to side effects in clinical settings. As a result, an automatic epileptic seizure detection system can diminish the pressure on the neurologists^7^.

Several algorithms have been introduced for epileptic seizure detection using EEG signals^8–10^. The raw EEG signal is often accompanied by noise and irrelevant information. To design a high-performance epileptic seizure detection system, preprocessing steps are crucial^11^. Salient and discriminative features have been extracted from signals thus far using popular machine learning algorithms, and their performance has been encouraging^12^. In all types of EEG signals, machine learning models indicate high performance^13^.

In the paper^14^, researchers have used empirical mode decomposition (EMD) and power spectral density of the resulting EMD components as features of seizure EEG signals. In^15^, the authors have introduced an efficient feature selection method, a Bagged Tree-based classifier (BTBC), and Explainable Artificial Intelligence (XAI) for the classification of epileptic EEG signals. In another study^16^, authors have used a Support Vector Machine to predict epileptic seizures in real-time. A paper^17^ has assessed the performance of different machine learning classifiers; thirteen discriminative features are evaluated and considered as inputs of the classifiers. In^18^, researchers have tested two distinct feature selection techniques—a data-based mathematical approach and expert domain knowledge of data (explainable features, or EFs)—to identify epileptic seizure EEGs. They have demonstrated that explainable features are better at classifying epileptic EEGs. A review paper^19^ has examined various state-of-the-art machine-learning techniques for predicting seizures using EEG signals. To enhance the effectiveness of machine learning-based detection systems, the paper^20^ proposes a framework for assessing the performance of both fuzzy-based and conventional machine learning-based models. It has been determined that KNN and its fuzzy variant achieve the best classification accuracy. In the article^21^, feature engineering has been performed using three types of features extracted from EEG signals in the time, frequency, and time-frequency domains. DWT is one of the feature extraction methods that can extract features from non-stationary and complex signals, such as EEG. In^22^, the DWT is used to extract frequency components from EEG signals, and the KNN method is employed to enhance detection performance. Several other studies have employed the DWT to decompose EEG signals^23–25^.

In recent studies, deep learning has been primarily used to process EEG signals and deep networks can be employed in various roles. Deep networks can be employed for EEG signal preprocessing steps such as noise removal or dimensionality reduction^26^. Autoencoders and other generative networks can be used for EEG preprocessing steps^27^. Deep networks can also be used as feature extractors; CNN, RNN, and Generative Adversarial Networks can be used for this purpose. Sometimes, these networks appear in the role of a classifier. Ultimately, the deep network is responsible for classifying EEG signals. There, deep networks carry out end-to-end learning, which includes the preprocessing phase extraction of features, selection of features, and classification all at once.

In work^28^, the authors have proposed a 1D CNN-LSTM model for seizure detection from EEG signals. In^25^, different bands of epileptic EEG are extracted using DWT, and the performance of several Random Neural Networks with different architectures is evaluated. CNN’s role as a classifier is investigated in^6^. Firstly, several features, such as Approximate Entropy (ApEn), Fuzzy Entropy (FuzzyEn), etc., of sub-bands of epileptic EEG signals are extracted. Then, to find the best feature set, the random forest is used, and the CNN is utilized for EEG classification. In^7^, Li et al. have proposed an end-to-end automatic seizure detection model that does not require heavy preprocessing. First, three convolution blocks are employed in the model architecture, and the recovered features are used as input to the Nested LSTM model. Classification is performed using the softmax layer. In^29^, Ma et al. have combined the Independent recurrent neural network (indRNN) and CNN to propose an automatic model for seizure detection. Initially, they utilized the residual architecture of a 1D CNN, and then the RNN network was used to learn the sequential features.

Researchers^30^ have combined the CWT and a CNN to propose an epileptic seizure detection system. In another study^31^, the DWT is employed for epileptic EEG denoising and the extraction of 20 eigenvalue features. Lastly, the LSTM network is used for epileptic EEG detection. The paper^5^ used a Deep neural network and Binary dragonfly algorithm (BDFA) to design an EEG-based automatic seizure detection model. In^32^, deep feature maps are extracted from hierarchical CNN layers, and dimension reduction is performed using PCA. Some shallow classifiers are used to detect anomalies. In the work^33^, the XAI4EEG model, which is a hybrid deep-learning model designed for detecting epileptic EEG, is introduced. In this paper, the authors consider deep learning models and domain knowledge in the context of seizure detection. In order to propose a model for automatic epileptic EEG detection, a paper^34^ combined a neural network classifier with a three-dimensional deep convolution autoencoder (3D-DCAE). In^35^, epileptic seizures are identified from EEG signals using a CNN network in conjunction with a patient-specific AE. A major challenge in using deep learning for EEG signal processing is the need for large datasets. In another study^36^, the authors explored the use of Generative Adversarial Networks (GANs) to enhance EEG signal data through augmentation.

Although recent works have used deep learning approaches for epileptic EEG signal recognition and DWT has also been used in the preprocessing steps to decompose the signal into different frequency bands, no study has used DWT and 1D CNN-LSTM simultaneously, where after extracting frequency bands from the EEG signal, the 1D CNN-LSTM network performs the detection operation. The deep learning architecture of some works has several parameters that are not optimal in real-time scenarios. Our proposed model applies to both multi-channel and single-channel EEG signals and ultimately converts these two types of signals into a one-dimensional feature vector, making it generalizable to different epileptic signals. Due to the dimensions of the feature vector extracted from DWT, one-dimensional CNN is used, which has fewer parameters than two-dimensional CNN. Our proposed model can be trained more efficiently compared to other deep learning-based models.

As evident from the literature, deep networks can be utilized as a high-performance detection model for epileptic seizures using EEG signals. Although some recent works have utilized raw EEG signals as input for deep networks, incorporating proper EEG features can enhance the performance of the detection model. DWT can be used to decompose the EEG signal into sub-bands. This motivated us to propose our model.

The following are the main contributions of this paper:

- A novel hybrid deep-learning architecture is proposed for the automatic classification of epileptic EEG signals.
- The proposed model applies to any type of input signal, whether single-channel or multi-channel.
- The performance of the novel method is compared with that of other standard classification algorithms, such as SVC and MLP.
- In terms of accuracy, precision, specificity, sensitivity, NPV, PPV, F1-score, and Matthews Correlation performance metrics, the presented model demonstrated superior efficiency in evaluating results.

This paper is organized as follows: First, in the Methods section, the data used for evaluating the proposed model are introduced. Second, the proposed detection system is explained in detail in the subsection “Proposed Model”. The model evaluation process and the experiments are presented in subsections. In the third section, the results of the performance metrics for the new model on the three benchmark datasets are presented. The Discussion section discusses the limitations of work and failed examples. In the fifth section, the work is concluded, and the future perspective for similar future research is introduced.

## Methods

### Datasets

Three publicly accessible datasets are used in this work. The first is the University of Bonn (BONN), and the second originates from the Children’s Hospital Boston-Massachusetts Institute of Technology (CHB-MIT). TUH EEG Seizure Corpus (TUSZ), a subset of the Temple University Hospital EEG Corpus, is the third dataset.

#### Bonn EEG dataset

The first dataset consists of the University of Bonn, Germany^37^ epilepsy EEG dataset, which was collected at the Department of Epileptology in Germany and includes recordings from both epileptic patients and healthy subjects. It is a single-channel EEG signal dataset that is divided into five subsets (Set A through Set E). 100 single-channel EEG data segments with a sample period of 23.6 s and a sampling rate of 173.61 Hz make up each subgroup. Each segment has roughly 4097 sample points, taking into account the period and sampling rate. The letters Z, O, N, F, and S stand for the subsets. Using the international 10/20 electrode system, the data was collected. Although the dataset provides only one channel per segment, the original recordings were multichannel, and the 10–20 system was used to guide electrode placement. Thus, the mention of the 10–20 system refers to the electrode configuration during acquisition, not to the dataset structure itself. There are five subsets (A-E) in Bonn EEG dataset with the following characteristics: Set A (Z) is sEEG (Surface EEG) from healthy subjects with open eyes; Set B (O) is sEEG from healthy subjects with closed eyes; Set C (F) is Intracranial EEG (iEEG) from epilepsy patients, hippocampus; Set D (N) is iEEG from epilepsy patients, epileptogenic zone; Set E (S) is iEEG from epilepsy patients with seizure, epileptogenic zone.

**Fig. 1:**
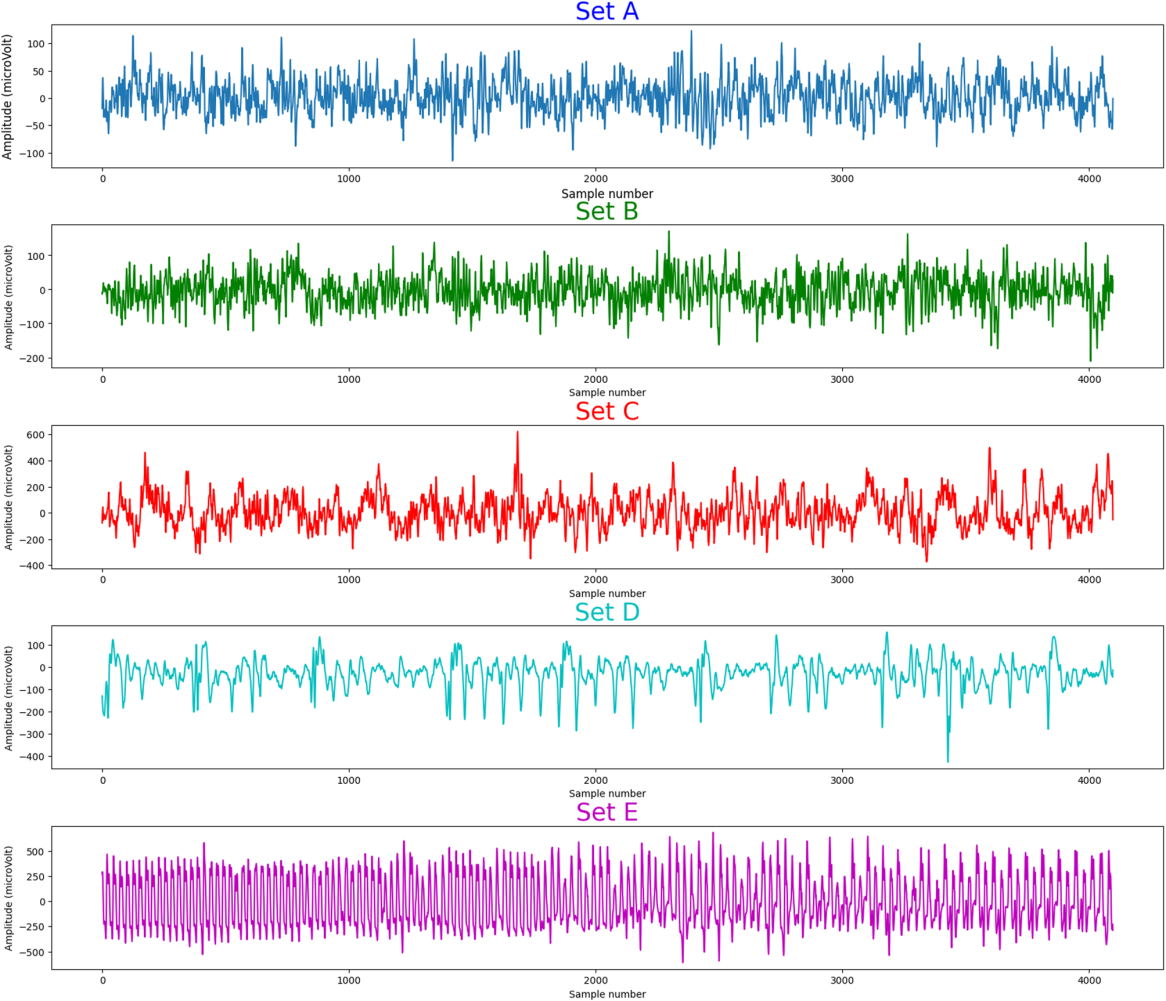
The Bonn EEG dataset contains five distinct kinds of epileptic EEG signals. A (Healthy, eyes open); B (Healthy, eyes closed); C (Epileptic, seizure-free (opposite hemisphere)); D (Epileptic, seizure-free (epileptogenic zone)), and E (Epileptic, during seizure (ictal) are the sets. The horizontal axis displays the sample number, and the vertical axis shows the amplitude.

Figure 1 shows the five different classifications.

#### CHB-MIT EEG dataset

Children’s Hospital Boston and the Massachusetts Institute of Technology (CHB-MIT) collaborated to create this dataset^38^. Raw EEG signals from 23 children with epilepsy diagnoses are included in the CHB-MIT database. Both males (ages 3–22 years) and females (ages 1.5–19 years) are included in the more than 980 h of data.

The EEG recordings are categorized into 24 groups and recorded in EDF data files, with a sampling frequency of 256 Hz and a 16-bit resolution for all patients. Remarkably, case chb21 was obtained from the same female subject 1.5 years after case chb01. However, recording times differ; some last an hour, while others last two or four hours. Additionally, each recording has a different number of channels; most of the recordings have 23 channels. In particular, seizure detection is done using the first 22 channels. About 846.23 h of EEG recordings, including 198 seizures, were analysed in this investigation.

**Fig. 2:**
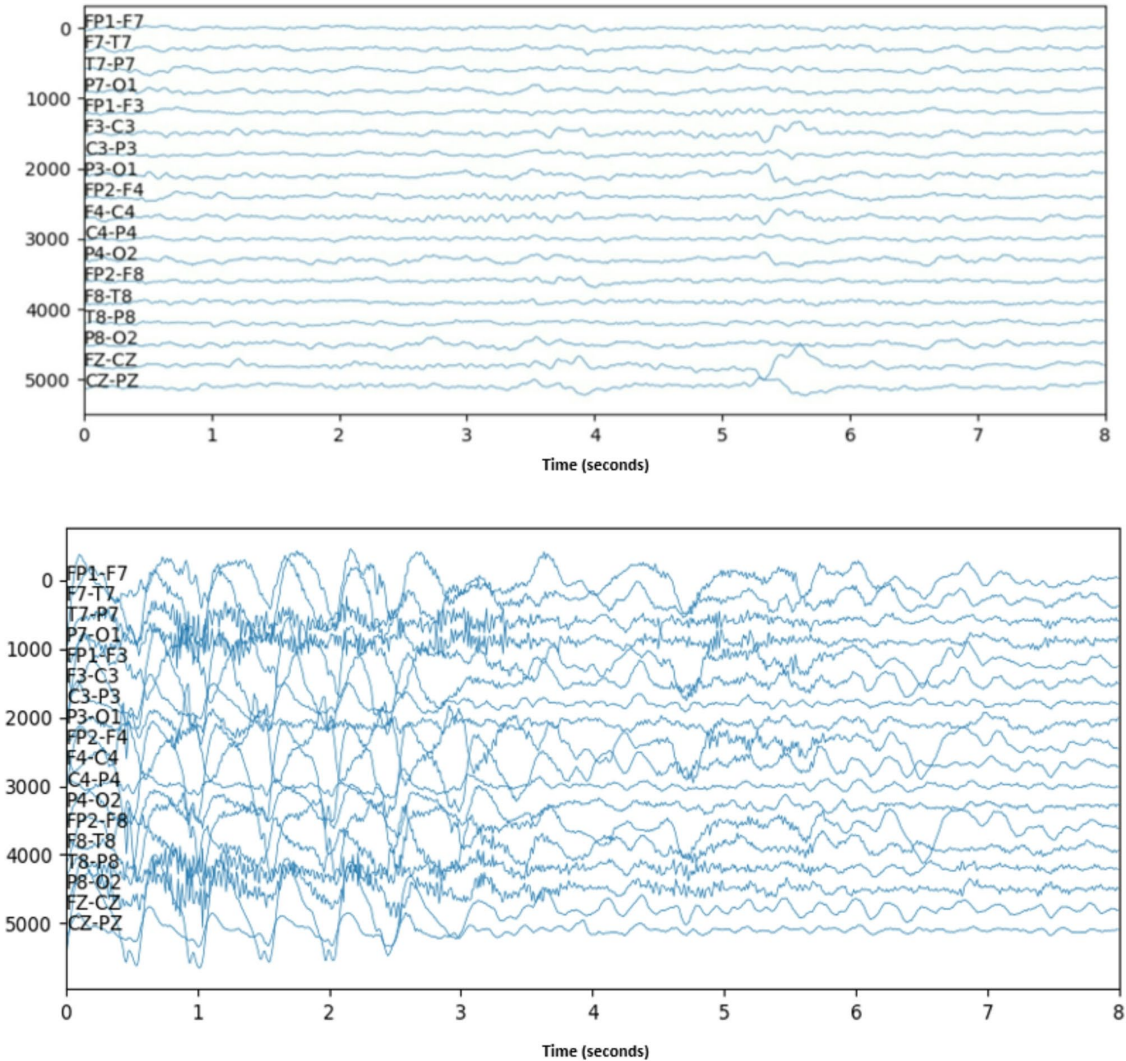
Raw EEG signal of the healthy subject (above) and epileptic seizure subject (bottom) of the CHB-MIT dataset. The vertical line indicates channels, and the horizontal line indicates time (s). Y-axis labels mean each channel is the difference between two electrodes. For example, channel 1 is the difference between electrodes FP1 and F7.

Figure 2 show an 8-second sample of raw EEG signals from both healthy subjects and those who were having epileptic seizures.

#### TUH EEG seizure corpus (TUSZ)

The Neural Engineering Data Consortium website (www.nedcdata.org) makes the Temple University Hospital (TUH) EEG Corpus publicly accessible^39^. We have used the Temple University Seizure Corpus (TUSZ), a subcategory of the TUH corpus, which is the most extensive EEG seizure corpus^40^. This corpus comprises both male and female subjects (51% women and 49% men), with an average age range of 1 to 90 years. Although 87% of the total data has a 250 Hz sampling frequency, the data has a frequency range between 250 and 1024 Hz. All the data is resampled to 256 Hz. The TUH EEG seizure data is recorded in standard 10–20 format, and it has 23 or 22 channels; we have considered 22 channels for all signals to construct a consistent dataset for our model. This dataset has been annotated with eight different seizure events by students and neurologists from Temple University. The eight different seizure types are categorized as: focal non-specific seizure (FNSZ), generalized non-specific seizure (GNSZ), simple partial seizure (SPSZ), complex partial seizure (CPSZ), absence seizure (ABSZ), tonic seizure (TNSZ), tonic-clonic seizure (TCSZ) and myoclonic seizure (MYSZ). The TUH team continuously updates the corpus, and it is one of the best seizure datasets for research purposes. The details of different types of seizures in this dataset are presented in Table 1^41^. We have constructed our EEG dataset array from this corpus using non-overlapping one-second signals (256 samples) with 22 channels.

**Table 1:** Description of different seizure classes in TUH EEG seizure corpus (TUSZ)^41^.

No.	Seizure type	Seizure description
1	FNSZ	Focal seizures which cannot be specified with its type
2	SPSZ	Only clinical indicators can identify partial seizures that occur while a person is conscious
3	CPSZ	Partial seizures during unconsciousness which is specified by clinical signs only
4	GNSZ	Generalized seizures which cannot be further specified with its type
5	ABSZ	Absence discharges observed on EEG where patient loses consciousness for few seconds (also known as petit mal)
6	TNSZ	Stiffening of body during seizure (EEG effects disappear)
7	TCSZ	At first stiffening and then jerking of body (also known as grand mal)
8	MYSZ	Myoclonous jerks of limbs

**Fig. 3:**
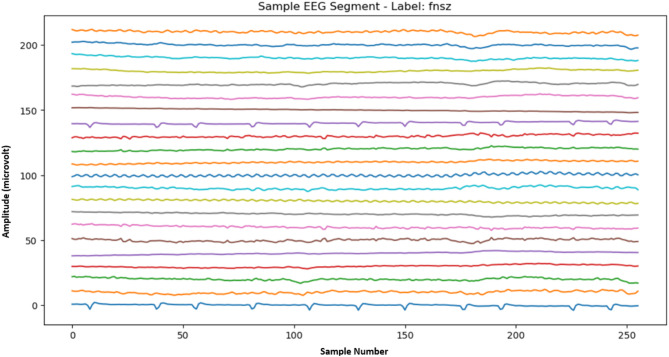
One second snapshot of FNSZ seizure class of TUSZ corpus. The vertical axis shows the Amplitude, and the horizontal axis shows the sample number.

Figure 3 illustrates a 1-second segment of FNSZ seizure class of TUSZ corpus.

### Proposed model

To design an efficient and high-performance model for detecting epileptic seizure EEG signals, we have examined various models. First, we have considered a case where the raw signal is fed into the CNN network and examined the model’s performance on three datasets. Then, the raw signal is processed using the RNN network and its family, as well as the AE network. We have assessed various feature extraction methods, including PCA, ICA, and DWT, and fed the output of these feature extractors to deep networks such as CNN and LSTM, which performed better than other deep learning networks. DWT extracts different frequency bands of the signal as some coefficients, and by concatenating its coefficients, a one-dimensional feature vector can be obtained. We have analyzed this feature vector with different deep networks and their combinations. For example, two-dimensional CNNs, one-dimensional CNNs, LSTM, BiRNN, GRU, and SimpleRNN. By comparing the results obtained, the best preprocessing and feature extractor was DWT, and the best 1D network was 1D CNN-LSTM, which performed best for all datasets.

#### Feature extraction using DWT

**Fig. 4:**
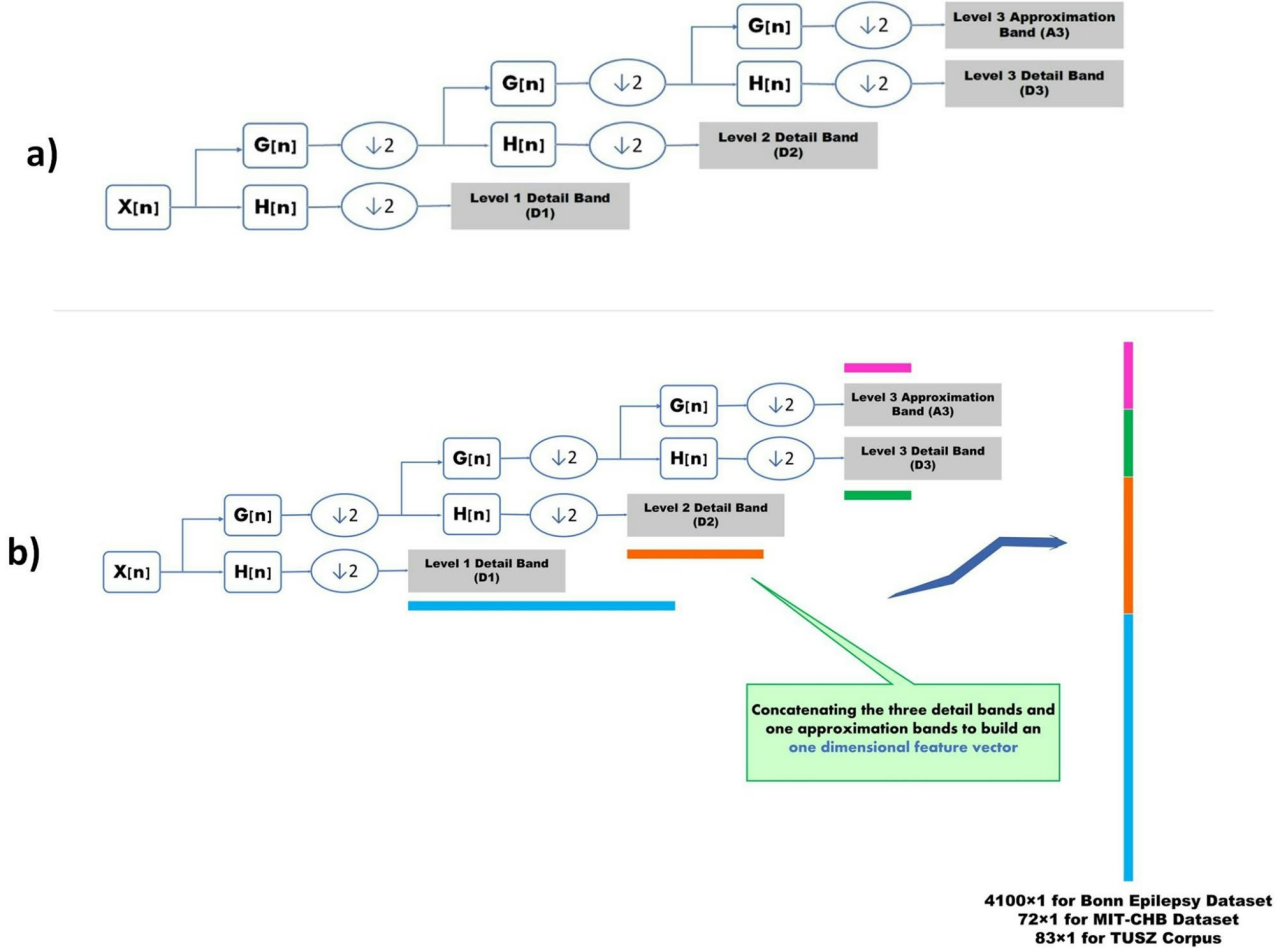
(**A**) EEG signal decomposition with 3-Level DWT. $\:\left(\downarrow\:2\right)\:$means down-sampling by half. (**B**) Concatenation of decomposed EEG signal using DWT to create a one-dimensional feature vector as an input for the 1D CNN-LSTM model. $\:\left(\downarrow\:2\right)\:$means down-sampling by half.

The DWT decomposes EEG signals into time-frequency components through multiresolution analysis^42,43^. The Discrete Wavelet Transform (DWT) uses a sequence of low-pass and high-pass filters to decompose the eeg signal into components.DWT generates two sets of coefficients at each level: approximation coefficients (ACs), which capture the low-frequency components, and detail coefficients (DCs), which are the high-frequency details. Unlike fixed-window methods, DWT does not segment the signal into equal time windows. Instead, it recursively filters and down samples the signal, yielding increasingly coarser approximations at each level. At each stage of decomposition, the approximation (low-frequency) part is further split into a new set of ACs and DCs. This hierarchical process continues based on the desired number of decomposition levels. The transformation is based on a selected mother wavelet, which determines the shape and characteristics of the basic functions used for analysis^44^. Daubechies-3 (DB-3) wavelet function is considered the mother wavelet that decomposes the raw EEG. Figure 4 illustrates DWT with a three-level Daubechies wavelet function. In the above part of Fig. 4, the approach of signal decomposition using DWT is illustrated. For multi-channel EEG signal, we first apply DWT separately to each EEG channel, then concatenate the resulting coefficients per channel into a 1D vector per channel, and finally stack these per-channel vectors to form a 2D array of shape (number of channels, coefficient length per channel) for each sample. Therefore, the DWT preserves channel-wise independence, allowing us to retain spatial locality and avoid mixing signals from different regions. On the other hand, the DWT is applied along the temporal axis of each EEG signal. We cannot concatenate coefficients across channels because this would mix temporal frequency features from different spatial origins.

After EEG signal decomposition, the extracted sub-bands are concatenated to create a long 1D vector as an input for the 1D CNN-LSTM model. The procedure of the 1D feature vector is shown in the bottom part of Fig. 4.

#### 1D CNN-LSTM model

**Fig. 5:**
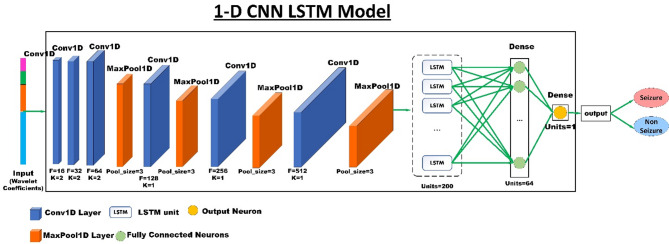
The proposed 1D CNN-LSTM model’s block diagram.

This section presents the suggested 1D CNN-LSTM model for classifying epileptic seizures and non-seizures (as well as for multi-class classification for various seizure types in the TUSZ corpus). The block diagram of the suggested model for binary classification is displayed in Fig. 5 (the same structure is used for multi-class classification in TUSZ, except the final dense layer, whose units should be increased by the number of classes). The 1D CNN and LSTM are used after the EEG signal has been decomposed using DWT.

Figure 5 shows the proposed 1D CNN-LSTM architecture, which consists of an input layer, six convolutional layers, four pooling layers, one LSTM layer, one fully connected (FC) layer, and a softmax output layer. The proposed model is fed the one-dimensional input, which is the concatenated coefficients of DWT, as an input. The input shape depends on the number of channels of the epilepsy EEG dataset. However, in both cases of multi-channel and one-channel signals, the input will be a one-dimensional feature vector because it is the result of the concatenation of some coefficients.

Due to the varying number of signal samples in each dataset and their single-channel or multi-channel nature, the level 3 DWT features that are merged will differ in each dataset, as shown in Fig. 5. Although all inputs to the model are one-dimensional, they have different lengths. The proposed architecture is flexible, and it does not matter what the exact dimensions of the one-dimensional vector input to it are; features are extracted in each layer, and finally, classification is performed. The first convolutional layer of the model is designed in such a way that it can use the input of any length and extract the appropriate feature from it. The first 1D convolutional layer has 16 filters with (2,1) kernel size and strides = 2 and padding=’valid’. Different length 1D vectors can be processed with this layer and subsequent layers. Therefore, the output dimensions and the number of parameters of each layer may differ for different datasets, but each convolutional layer extracts the appropriate spatial features. Ultimately, the LSTM layer extracts the appropriate temporal features and performs well across all three datasets.

Subsequently, the input features are fed into the first convolutional layer to extract general features from the input. In this layer, the number of learned kernels is 16, each with a $\:2\times\:1\:$kernel size, the stride of convolutional kernels is 2, and padding is valid for each of the convolutional layers. After each convolutional layer, there is a batch normalization layer to reduce the probability occurrence of overfitting. Batch normalization standardizes the entire batch by centering it around zero.

During the normalization, the mean (µ) and variance ($\:{\sigma\:}^{2}$) computed within a batch need to be estimated:

1 $$\:\mu\:=\frac{1}{b}\sum\:_{i=1}^{b}{X}^{\left(i\right)}$$

2 $$\:{\sigma\:}^{2}=\frac{1}{b}\sum\:_{i=1}^{b}{(X}^{\left(i\right)}-\mu\:{)}^{2}$$

In Eqs. (1) and (2), in each batch, the number of training data is b, and $\:{X}^{\left(i\right)}$ is a training data. Equation (3) computes the zero-centered normalized value of $\:{X}^{\left(i\right)}$:

3 $$\:{\widehat{X}}^{\left(i\right)}=\frac{{X}^{\left(i\right)}-\mu\:}{\sqrt{{\sigma\:}^{2}+\xi\:}}\:,\:\:\xi\:={10}^{-5}$$

Equation (4) is used for scaling and offsetting where $\:\gamma\:$ represents the scaling parameter, and $\:\beta\:$ denotes the offset parameter. The operator $\otimes$ is an element-wise multiplication.

4 $$\:z^{i} = \gamma \: \otimes \hat{X}^{{\left( i \right)}} + \beta \:$$

The mathematical expression for a one-dimensional convolutional operation with ReLU activation function is presented in Eq. (5):

5 $$\:{y}_{j}^{l}=\sigma\:\left(\sum\:_{i=1}^{{N}_{l-1}}conv1D\left({K}_{i,j}^{l},\:{x}_{i}^{l-1}\right)+{b}_{j}^{l}\right)$$

Where $\:{x}_{i}^{l-1}$ is the $\:i$th feature map in the $\:(l-1)$th layer, $\:{y}_{j}^{l}$ indicates the $\:j$th output feature map in $\:l$th layer, $\:{K}_{i,j}^{l}$ is the convolutional kernel, $\:{N}_{l-1}$ indicates the number of feature maps in the layers, $\:{b}_{j}^{l}$ is the bias of $\:j$th feature map in $\:l$th layer, and $\:conv1D$ is the 1D convolution operation.

After each convolutional layer, ReLU activation function is used. $\:\sigma\:\left(\right)$ is the ReLU activation function, which is used to suppress overfitting and increase the non-linearity of the model. Equation (6) shows this activation function:

6 $$\:\sigma\:\left(x\right)=\:\left\{\begin{array}{c}0,\:\:\:\:x\le\:0\\\:x,\:\:\:\:\:\:x>0\end{array}\:\right.$$

After the first convolutional layer, the second convolutional layer with 32 filters with a size of 2 × 1 is used. In the following, four convolutional blocks (comprising Conv1D, batch normalization, ReLU activation function, and max pooling1D) are employed to enable the model to extract finer and more informative features. The resulting feature map is given to a single LSTM layer with 200 neurons. The inner structure of the LSTM layer will be explained in the following sections. In the context of multiple-class classification, the softmax activation function is applied to derive the probabilities for each class:

7 $$\:{\widehat{y}}_{i}=argmax\left(\frac{{e}^{{y}_{i}}}{{\sum\:}_{i=1}^{N}{e}^{{y}_{i}}}\right)$$

The detailed structure of the proposed model will be adjusted based on the specific requirements for epileptic seizure detection and the number of classes. Compared to other optimizers, Adam is particularly well-suited for high-dimensional data. Adam utilizes a mini-batch stochastic gradient optimization method. The cross-entropy loss function is used to train the proposed one-dimensional CNN-LSTM model with a learning rate of $\:{10}^{4}$. The layers of the proposed model are detailed in Table 2, which also shows the output shape and parameters of each layer. Binary cross-entropy is used for binary classification scenarios, while categorical cross-entropy is used for multi-class cases.

**Table 2:** Model summary of the proposed 1D CNN-LSTM model.

No.	Layer name	Layer parameters	Output shape	Number of parameters
1	1D convolution	Filters = 16, kernel_size = (2,1) input_shape = (4100,1), strides = 2, padding = ‘valid’	(2050,16)	48
2	BatchNorm		(2050,16)	64
3	Activation	ReLU	(2050,16)	0
4	1D Convolution	Filters = 32, kernel_size = (2,1), strides = 2, padding = ‘valid’	(1025,32)	1056
5	BatchNorm		(1025,32)	128
6	Activation	ReLU	(1025,32)	0
7	1D Convolution	Filters = 64, kernel_size = (2,1), strides = 2, padding = ‘valid’	(512,64)	4160
8	BatchNorm		(512,64)	256
9	Activation	ReLU	(512,64)	0
10	MaxPool1D	Pool_size = 3, strides = 2, padding=” valid”	(255,64)	0
11	1D Convolution	Filters = 128, kernel_size = (1,1), strides = 1, padding = ‘valid’	(255,128)	8320
12	BatchNorm		(255,128)	512
13	Activation	ReLU	(255,128)	0
14	MaxPool1D	Pool_size = 3, strides = 2, padding=” valid”	(127,128)	0
15	1D Convolution	Filters = 256, kernel_size = (1,1), strides = 1, padding = ‘valid’	(127,256)	33,024
16	BatchNorm		(127,256)	1024
17	Activation	ReLU	(127,256)	0
18	MaxPool1D	Pool_size = 3, strides = 2, padding=” valid”	(63,256)	0
19	1D Convolution	Filters = 512, kernel_size = (1,1), strides = 1, padding = ‘valid’	(63,512)	131,584
20	BatchNorm		(63,512)	2048
21	Activation	ReLU	(63,512)	0
22	MaxPool1D	Pool_size = 3, strides = 2, padding=” valid”	(31,512)	0
23	LSTM	Units = 200	200	570,400
24	Flatten		200	0
25	Dense	Unit = 64, activation = “ReLU”, kernel_regularizer = *L*2 (0.03)	64	12,864
26	Dropout	Rate = 0.4	64	0
27	Dense	Unit = 1	1	65
28	Activation	sigmoid	1	0
Total				765,553

##### 1D CNN

Regardless of whether our input signal is single-channel or multi-channel, the feature vector will be one-dimensional, which is an advantage because the model can apply to both types of datasets. The 1D CNN, which utilizes 1D convolution operations with multiple filters and one-dimensional max pooling, effectively extracts important and meaningful representations from EEG signals. By using a deep architecture of the CNN network, it will be possible to extract higher-level feature maps gradually. To design an automatic epileptic seizure detection system, a robust and discriminative feature set will be needed.

##### LSTM structure

**Fig. 6:**
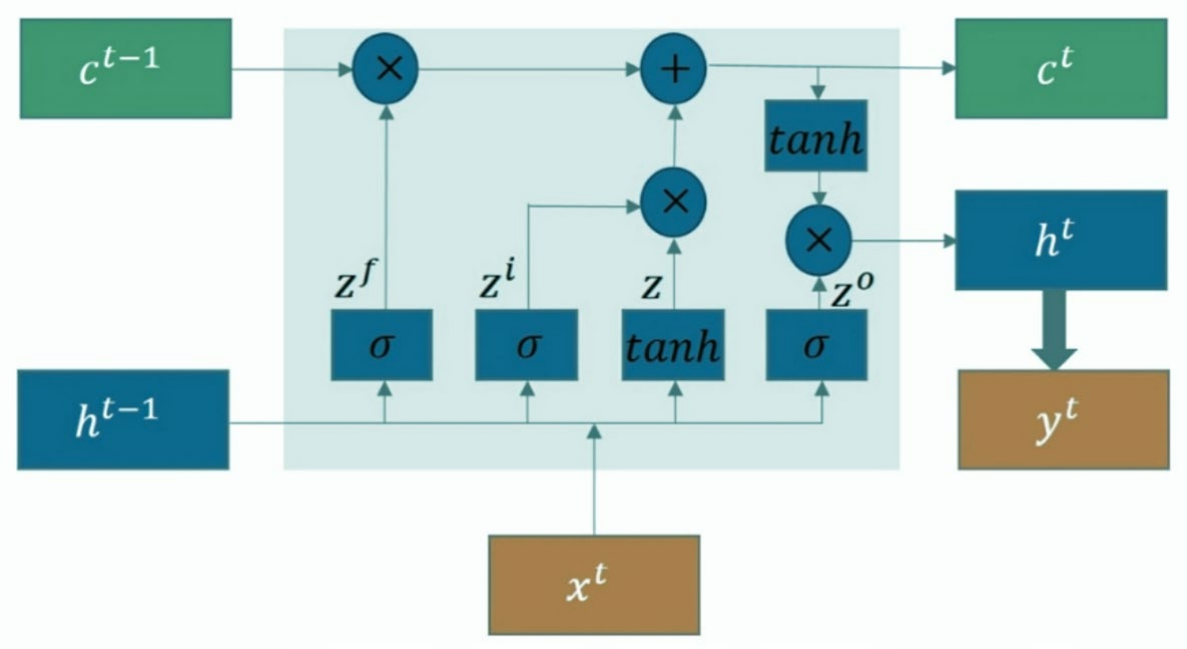
The internal Structure of the LSTM layer^28^.

The LSTM network is one of most significant networks in the RNN family. LSTM can memorize long-term dependencies, which can be an advantage for time series data like EEG signals. Figure 6 depicts the LSTM block’s internal organization. There are four main gates in the LSTM block, which are cell state gate $\:z$, forget gate $\:{z}^{f}$, input gate $\:{z}^{i}$, and output gate $\:{z}^{o}$. Cell state gate characterizecha how much information should be remembered from past. Forget gate the extent of information that should be kept in the cell. The input gate governs the extent to which new data influences the cell’s internal state, while the output gate determines the information used for output. Within a LSTM block, three inputs exist: the cell state $\:{c}^{t-1}$, the previous hidden state $\:{h}^{t-1}$, and the current input $\:{x}^{t}$. There are also three outputs, which are cell state $\:{c}^{t}$, hidden state $\:{h}^{t-1}$, and current output $\:{y}^{t}$. The mathematical equations used to calculate each of these quantities are provided below.

8 $$\begin{aligned} & z^{f} = \sigma \:\left( {W^{f} \left[ {x_{t} \:,\:h_{{t - 1}} } \right]} \right) \\ & z^{i} = \sigma \:\left( {W^{i} \left[ {x_{t} \:,\:h_{{t - 1}} } \right]} \right) \\ & z = tanh\left( {W\left[ {x_{t} \:,\:h_{{t - 1}} } \right]} \right) \\ & z^{o} = \sigma \:\left( {W^{o} \left[ {x_{t} \:,\:h_{{t - 1}} } \right]} \right) \\ & c^{t} = z^{o} \times \:tanh\left( {c^{t} } \right) \\ & y^{t} = \sigma \:\left( {W\prime \:h_{t} } \right) \\ \end{aligned}$$

To compare the proposed model with other possible deep networks, an RNN network with an attention mechanism is also used. That is, without using a 1D CNN network at the beginning of the model, only an RNN with a different number of layers (for performance comparison) is used with the attention mechanism. Although the RNN with attention model was evaluated on three datasets—Bonn, CHB-MIT, and TUSZ—it performed inferiorly to the proposed model in this study.

#### Machine learning classifiers

We have conducted several experiments to demonstrate the superiority of our proposed model over conventional machine learning classifiers. The classifiers that are employed include support vector classifier (SVC)^45^, K-nearest neighbor (KNN)^46^, Gaussian Naïve Bayes (GNB)^47^, decision tree (DT)^48^, and multi-layer perceptron (MLP)^49^. These models served as benchmark models for comparison versus our proposed 1D CNN-LSTM model; This approach highlights the capabilities of the model.

### Method evaluation

Our experiments are carried out using Google Colab Pro with a TPU backend, using 35 GB RAM and 225 GB Disk RAM to train and evaluate models in order to evaluate the effectiveness of the suggested seizure detection model. The TensorFlow framework of python is used to create these models.

### Experimental setup

We used 10-fold cross-validation as it provides a suitable balance between training bias and test variance^50^. We have change the number of convolutional and LSTM layers, optimiztion functions, learning rates, batch sizes and epochs to find the optimal model. Different filter sizes and parameters are tested, with epoch values ranging from 50 to 400. Epochs less than 300 yield lower accuracy, while those over 300 do not significantly improve performance but increased computational cost. Learning rates above 0.0001 slowed convergence, and smaller rates sometimes prevented convergence. Despite using Google Cloud, increasing epochs made training more expensive and time-consuming. The model with best performance was with one LSTM layer (200 neurons), Adam optimization, a learning rate of 0.0001, batch size of 60, 300 epochs, and a validation split of 0.15.

To evaluate the model, a 10-fold cross-validation approach is used, with 90% of data for training and 10% for testing. From the training data, 15% is further split for validation, resulting in 76.5% training, 13.5% validation, and 10% testing per fold. This method ensured unbiased performance evaluation with an independent test set. A 15% validation split is chosen for a balanced trade-off between training and performance monitoring, leading to more stable validation accuracy and loss curves. This approach helps reduce overfitting by maintaining a large and diverse validation set while providing sufficient data for training.

Various pre-processing experiments are conducted on each dataset, analysing wavelet functions like db, haar, and coif at different decomposition levels. The db1 wavelet function with three decomposition levels is found to be the best. Dropout is used to improve generalization and prevent overfitting. The data is randomly shuffled before training and at the end of each epoch. To evaluate the model’s performance and ensure generalization, accuracy and loss are calculated during training on both the training and testing datasets.

The EEG Bonn dataset includes five subsets (A–E). Sets A and B feature EEG recordings from healthy individuals with eyes open and closed, respectively. Sets C, D, and E are recorded from epileptic patients, with sets C and D during non-seizure periods, and set E during seizure episodes. This dataset supports both binary and multi-class classification tasks. All different cases for Bonn epilepsy dataset are shown in Table 3. The CHB-MIT dataset is used for binary classification, distinguishing between seizure and non-seizure EEG segments. The TUSZ corpus supports both binary and multi-class classification, involving different seizure types.

The complete code for pre-processing, training, and evaluation scripts, along with environment specifications, is available in the public GitHub repository: [https://github.com/Homakashefi/1D-CNN-LSTM-for-Seizure-Detection-EEG-Signals-/tree/main]. This repository contains all necessary scripts for replicating the experiments presented in this study.

**Table 3:** Different experimental cases for Bonn epilepsy EEG dataset.

Case	Group	Type 1	Type 2	Type 3	Type 4	Type 5
1	A vs. E	Set E (iEEG, epileptic, seizure)	Set A (sEEG, healthy, eyes open)			
2	B vs. E	Set B (sEEG, healthy, eyes closed)	Set E (iEEG, epileptic, seizure)			
3	AB vs. E	Set AB (sEEG, healthy)	Set E (iEEG, epileptic, seizure)			
4	C vs. E	Set C (iEEG, epileptic, interictal, hippocampus)	Set E (iEEG, epileptic, seizure)			
5	D vs. E	Set D (iEEG, epileptic, interictal, epileptogenic zone)	Set E (iEEG, epileptic, seizure)			
6	CD vs. E	Set CD (iEEG, epileptic, interictal)	Set E (iEEG, epileptic, seizure)			
7	A vs. D	Set A (sEEG, healthy, eyes open)	Set D (iEEG, epileptic, interictal)			
8	ABCD vs. E	Sets ABCD (non-seizure, sEEG + iEEG)	Set E (iEEG, epileptic, seizure)			
9	AB vs. CDE	Set AB (sEEG, healthy)	Sets CDE (iEEG, epileptic, mixed)			
10	A vs. C vs. E	Set A (sEEG, healthy, eyes open)	Set C (iEEG, epileptic, interictal)	Set E (iEEG, epileptic, seizure)		
11	AB vs. CD vs. E	Set AB (sEEG, healthy)	Set CD (iEEG, epileptic, interictal)	Set E (iEEG, epileptic, seizure)		
12	A vs. B vs. C vs. D vs. E	Set A (sEEG, healthy, eyes open)	Set B (sEEG, healthy, eyes closed)	Set C (iEEG, interictal, hippocampus)	Set D (iEEG, interictal, epileptogenic zone)	Set E (iEEG, seizure)

## Results

We have designed comprehensive experiments on three datasets. The aim is to illustrate the proposed model’s best performance in detecting epileptic EEG signals in two frameworks: binary and multi-class classification.

Other evaluation criteria, including specificity (SPF), sensitivity (SEN), negative predictive value (NPV), positive predictive value (PPV), F1 score, and Matthew’s correlation coefficient (MCC), have been employed in addition to accuracy (ACC) to assess the model. The Eqs. (9–15) for each metric are displayed below:

The following symbols are used to signify the True Positive (TP), False Negative (FN), True Negative (TN), and False Positive (FP):

9 $$\:SEN=\frac{TP}{TP+FN}\times\:100\left(\%\right)$$

10 $$\:SPF=\frac{TN}{TN+FP}\times\:100\left(\%\right)$$

11 $$\:ACC=\frac{TP+TN}{TP+TN+FN+FP}\times\:100\left(\%\right)$$

12 $$\:PPV=\frac{TP}{TP+FP}\times\:100\left(\%\right)$$

13 $$\:NPV=\frac{TN}{TN+FN}\times\:100\left(\%\right)$$

14 $$\:MCC=\frac{TP\times\:TN-FN\times\:FP}{\sqrt{(TP+FN)(TP+FP)(TN+FN)(TN+FP)}}\times\:100\left(\%\right)$$

15 $$\:F1\:score=\frac{2\times\:TP}{2\times\:TP+FN+FP}\times\:100\left(\%\right)$$

The results of the suggested model and additional classifiers on different categorization scenarios of the Bonn EEG dataset are shown in Table 4. Table 4 contains the average of all twelve cases. The assessment metrics of each fold for the suggested model can be found as Supplementary Table S1. The new model’s performance has been evaluated against that of various classifiers, including SVC, KNN, GNB, DT, and MLP.

**Table 4:** Average performance metrics for 10-fold cross validation of the proposed model and machine learning classifiers on twelve different cases of the Bonn EEG dataset.

Model (Mean ± SD)	Accuracy	Precision	Specificity	Sensitivity	NPV	PPV	F1-score	Matthews correlation
SVC	84.34 ± 0.83%	93.05 ± 0.74%	98.11 ± 0.61%	77.12 ± 0.81%	81.19 ± 0.91%	93.17 ± 0.86%	82.18 ± 0.86%	72.22 ± 1.14%
KNN	61.23 ± 0.86%	74.82 ± 0.91%	86.55 ± 0.63%	78.61 ± 0.76%	83.03 ± 0.89%	78.33 ± 0.89%	89.08 ± 0.85%	81.50 ± 0.93%
GNB	82.72 ± 0.63%	89.72 ± 0.63%	98.22 ± 0.39%	76.39 ± 0.69%	82.62 ± 0.62%	89.00 ± 0.74%	78.01 ± 0.55%	68.16 ± 0.77%
DT	78.52 ± 0.49%	79.54 ± 0.69%	89.34 ± 0.52%	73.53 ± 0.69%	77.06 ± 0.55%	79.50 ± 0.64%	74.14 ± 0.55%	78.00 ± 0.76%
MLP	72.01 ± 0.51%	69.45 ± 0.71%	78.23 ± 0.61%	75.07 ± 0.77%	74.73 ± 0.67%	68.57 ± 0.76%	79.13 ± 0.61%	81.02 ± 0.68%
Proposed model	**97.24 ± 0.38%**	**96.73 ± 0.51%**	**99.19 ± 0.34%**	**94.00 ± 0.47%**	**97.10 ± 0.53%**	**99.02 ± 0.40%**	**95.17 + 0.50%**	**93.03 ± 0.56%**

The best performances are in bold.

To evaluate the generalizability of our proposed model, the CHB-MIT dataset, which includes the multi-channel data of 24 subjects, is used. The average performance metrics for the proposed model and each ML classifiers are given in Table 5. For each fold, the proposed model’s performance and other machine learning classifiers for all the subjects can be found in Supplementary Table S2.

**Table 5:** Average performance metrics for 10-fold cross-validation of the proposed model and machine learning classifiers on all subjects of the CHB-MIT dataset.

Model (Mean ± SD)	Accuracy	Precision	Specificity	Sensitivity	NPV	PPV	F1-score	Matthews correlation
SVC	89.28 ± 1.14%	86.83 ± 1.53%	94.01 ± 1.71%	61.02 ± 0.85%	89.46 ± 1.84%	83.55 ± 1.76%	66.24 ± 1.37%	65.37 ± 0.75%
KNN	82.96 ± 0.70%	76.64 ± 1.23%	94.66 ± 1.54%	39.42 ± 1.27%	82.42 ± 0.84%	73.79 ± 0.90%	46.59 ± 0.97%	42.88 ± 1.55%
GNB	81.60 ± 1.52%	62.19 ± 1.58%	85.31 ± 1.50%	62.91 ± 1.14%	84.91 ± 1.55%	57.54 ± 1.73%	59.32 ± 2.53%	48.94 ± 0.64%
DT	91.06 ± 0.30%	91.50 ± 0.74%	92.68 ± 0.76%	92.12 ± 0.68%	90.56 ± 0.66%	87.00 ± 0.71%	89.30 ± 0.63%	84.92 ± 0.75%
MLP	92.11 ± 1.18%	91.14 ± 1.21%	93.19 ± 1.28%	92.36 ± 1.22%	95 ± 1.25%	94.76 ± 1.34%	86.06 ± 1.04%	85.77 ± 1.15%
Proposed model	**96.94 ± 1.22%**	**95.43 ± 1.23%**	**98.12 ± 1.28%**	**92.21 ± 1.17%**	**96.83 ± 1.20%**	**95.30 ± 1.29%**	**93.31 ± 1.05%**	**91.15 ± 1.12%**

The best performances are in bold.

To consider a different seizure dataset with different classes for seizure types, the TUSZ corpus is used. There are 53 subjects in dev (test) folder of this dataset. The number of classes of seizure types in each subject is different. So, this corpus can be considered both as binary and multi-class classification paradigms. The average performance of proposed model (multi-class classification) for all 53 subjects and its performance with other machine learning classifiers is illustrated in Table 6. Per-fold evaluation metrics are presented as Supplementary Table S3.

**Table 6:** Performance metrics of the proposed model and machine learning classifiers on all subjects of the TUSZ corpus.

Model (Mean ± SD)	Accuracy	Precision	Specificity	Sensitivity	NPV	PPV	F1-score	Matthews correlation
SVC	81.23 ± 1.19%	79.45 ± 1.21%	87.67 ± 1.35%	82.60 ± 1.28%	91.23 ± 1.15%	81.23 ± 1.24%	77.23 ± 1.32%	84.32 ± 1.14%
KNN	91.75 ± 1.20%	80.21 ± 1.19%	93.56 ± 1.35%	81.34 ± 1.28%	92.90 ± 1.22%	77.56 ± 1.24%	74.51 ± 1.32%	81.47 ± 1.15%
GNB	83.56 ± 1.18%	79.34 ± 1.20%	91.23 ± 1.30%	79.58 ± 1.25%	90.30 ± 1.19%	75.76 ± 1.22%	81.25 ± 1.29%	83.45 ± 1.18%
DT	92.34 ± 1.23%	88.65 ± 1.22%	94.56 ± 1.30%	81.25 ± 1.28%	94.32 ± 1.21%	80.56 ± 1.24%	85.10 ± 1.29%	83.79 ± 1.16%
MLP	89.95 ± 1.22%	82.65 ± 1.23%	83.72 ± 1.28%	82.37 ± 1.25%	95.91 ± 1.19%	75.95 ± 1.22%	83.79 ± 1.26%	85.50 ± 1.18%
Proposed model	**94.32 ± 0.86%**	**89.81 ± 0.87%**	**98.26 ± 0.88%**	**83.62 ± 0.86%**	**97.25 ± 0.89%**	**79.81 ± 0.84%**	**84.52 ± 0.87%**	**86.39 ± 0.85%**

The best performances are in bold.

As shown in Tables 4, 5 and 6, the performance metrics of the proposed model in three benchmark datasets are higher than other ML classifiers and it illustrates the effectiveness of our model in different epilepsy seizure datasets. In this way, we can claim that the performance of our proposed model is not only dependent on a specific epilepsy dataset or specific conditions, but is effective for different epileptic seizure datasets and can be used as a model in clinical epilepsy applications.

To address class imbalance in the CHB-MIT dataset, we employed stratified k-fold cross-validation and used a class-weighted loss function. Beyond standard metrics such as accuracy and Kappa, we report class-wise precision, recall, F1-score, and AUC-ROC. The model indicates strong discriminative performance even on the minority seizure class. The detailed per-class metrics for CHB-MIT dataset can be found as Supplementary Table S4. From this table, it can be seen that for both seizure and non-seizure classes, the performance metrics are high. Although the seizure cases in CHB-MIT are less than non-seizure cases, but we can see just little difference between the performance metrics of the cases.

While our model demonstrates strong overall performance, the clinical implications of even a low false positive rate—such as the 0.81% observed on the Bonn dataset—must be carefully considered. In emergency and critical care settings, false positives could lead to unnecessary interventions, increasing patient risk and healthcare burden. To reduce false alarms, the system can flag uncertain detections for expert review using confidence thresholds. Combining EEG with video monitoring further strengthens reliability through a multimodal approach^51,52^. Video monitoring captures behavioral and motor manifestations of seizures, such as convulsions or automatisms, which may not always be discernible in EEG alone. When EEG data is paired with video footage, doctors can see exactly how brain activity relates to what is happening in the body. This makes it much easier to tell real seizures from false alarms or other non-seizure events. As a result, diagnoses become more accurate, and doctors can act faster and with greater confidence when every second counts.

The model achieves high F1 scores across all seizure types, ranging from 77.53% (TNSZ) to 86.54% (GNSZ), with overall accuracy above 91% for all classes (see Supplementary Table S5 online). It demonstrates robustness, performing well even on rare seizure types like ABSZ and TNSZ. CPSZ consistently outperforms other classes in Matthews Correlation, F1-score, Precision, Accuracy, and AUC-ROC. TNSZ shows the lowest performance, particularly in sensitivity and AUC-ROC. The model’s high specificity and NPV across all datasets indicate strong ability to minimize false positives, which is crucial for clinical applications. These results suggest that CPSZ is easier to classify, while TNSZ may need further optimization.

Confusion matrices for each seizure class were estimated from class-wise sensitivity, specificity, and precision metrics combined with known class sample sizes in the TUSZ dataset. These matrices approximate model performance in a one-vs-rest evaluation setting, providing insights into class-wise detection characteristics (see Supplementary Fig. S1 online). Although it is difficult to classify different types of epileptic seizures, given that they may have common features, our proposed model was able to classify each of the different classes of epileptic seizures with high accuracy.

The best fit which is the goal of each learning algorithm and is between overfit and underfit model. A good fit is where training and validation loss decrease to a point of stability and there is also a minimal gap between training and validation losses. Also, the training loss of the model is always lower than validation loss. In other words, we expect there will be a small gap between training and validation loss curves which is called generalization gap. There is the same situation for this proposed model and datasets. Loss and accuracy plots for datasets can be found as Supplementary Fig. S2 and Fig. S3.

**Fig. 7:**
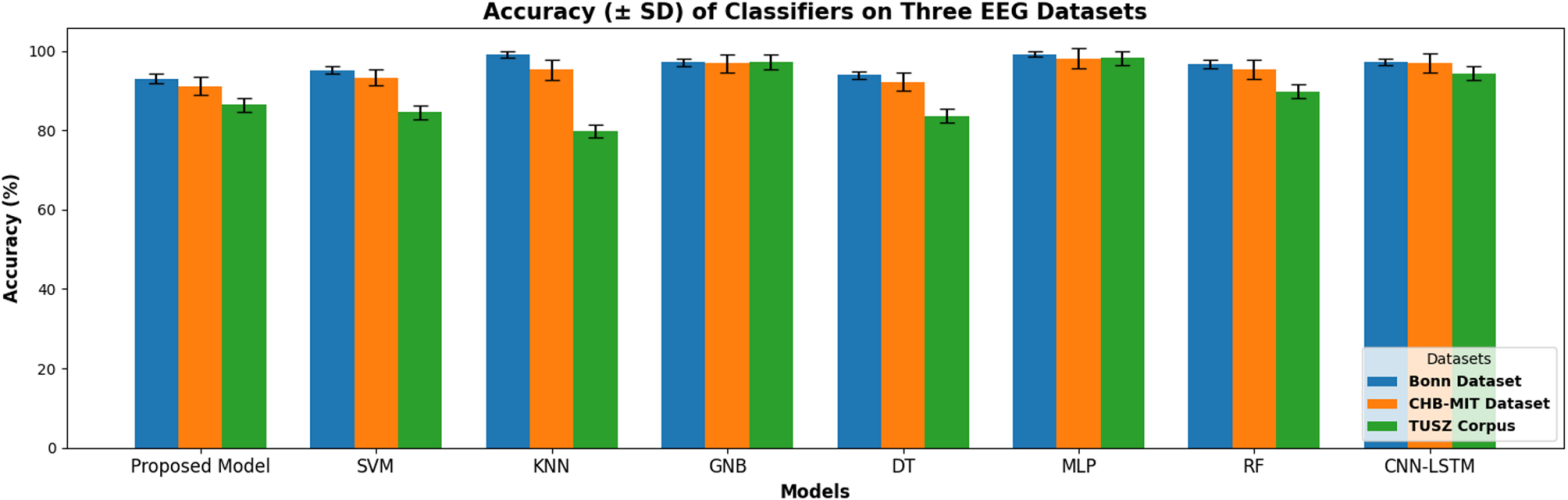
Bar accuracy plot of the proposed network and other machine learning classifiers on Bonn and CHB-MIT and TUSZ datasets.

In Fig. 7, bar plots compare the proposed model’s accuracy with other classifiers for two datasets: Bonn and CHB-MIT. The proposed network achieves the best accuracy in both datasets.

AUC-ROC is a performance metric which is used to evaluate binary classification models and it can also be used for multi-class scenarios. For binary scenarios (Bonn epilepsy and CHB-MIT datasets), the AUC values of 1.0 indicates a great ability of the proposed model to discriminate between seizure and non-seizure classes and for multi-class scenarios (Bonn epilepsy and TUSZ datasets), the closeness of plots to left-up corners show the higher area under the curve for each class, as a result the best case for each class of seizure types.

AUC-ROC plots for three datasets of Bonn epilepsy, CHB-MIT and TUSZ can be found as Supplementary Fig. S4 and Fig. S5 and Fig. S6.

In this study, we use DWT to capture the temporal patterns within each EEG channel. DWT breaks down the time-series signal into multiple frequency bands, preserving both when and at what frequency-specific patterns occur. It is applied separately to each channel, and the resulting wavelet features are then combined across channels to create the final input. This approach keeps the unique temporal dynamics of each channel intact while remaining flexible enough to handle both single-channel and multi-channel EEG data. While the model does not explicitly capture spatial relationships between electrodes—such as how signals from different locations interact—it still maintains some spatial context through the organized structure of its features. This makes the model adaptable to various EEG setups, whether they use a few electrodes or many.

We have compared the performance of our proposed model with baseline classifiers (SVM, KNN, GB, etc.) using a paired Student’s t-test on results from k-fold cross-validation (k = 10). We evaluated our model’s performance against five commonly used baseline classifiers: SVC, KNN, GNB, DT and MLP using 10-fold cross-validation. To assess the significance of the results, we conducted paired Student’s *t*-tests on fold-wise accuracy scores. All comparisons showed statistically significant differences, with large effect sizes: SVC (*p* = 0.003, *d* = 0.89), KNN (*p* = 0.001, *d* = 1.02), GNB (*p* < 0.001, *d* = 1.25), DT (*p* = 0.005, *d* = 0.84), and MLP (*p* = 0.002, *d* = 0.95). To further ensure the robustness of these findings, we also applied the Wilcoxon signed-rank test, which confirmed statistically significant differences across all comparisons (*p* < 0.01). Together, these results clearly show that our model consistently and significantly outperforms traditional classifiers in seizure classification tasks.

**Fig. 8:**
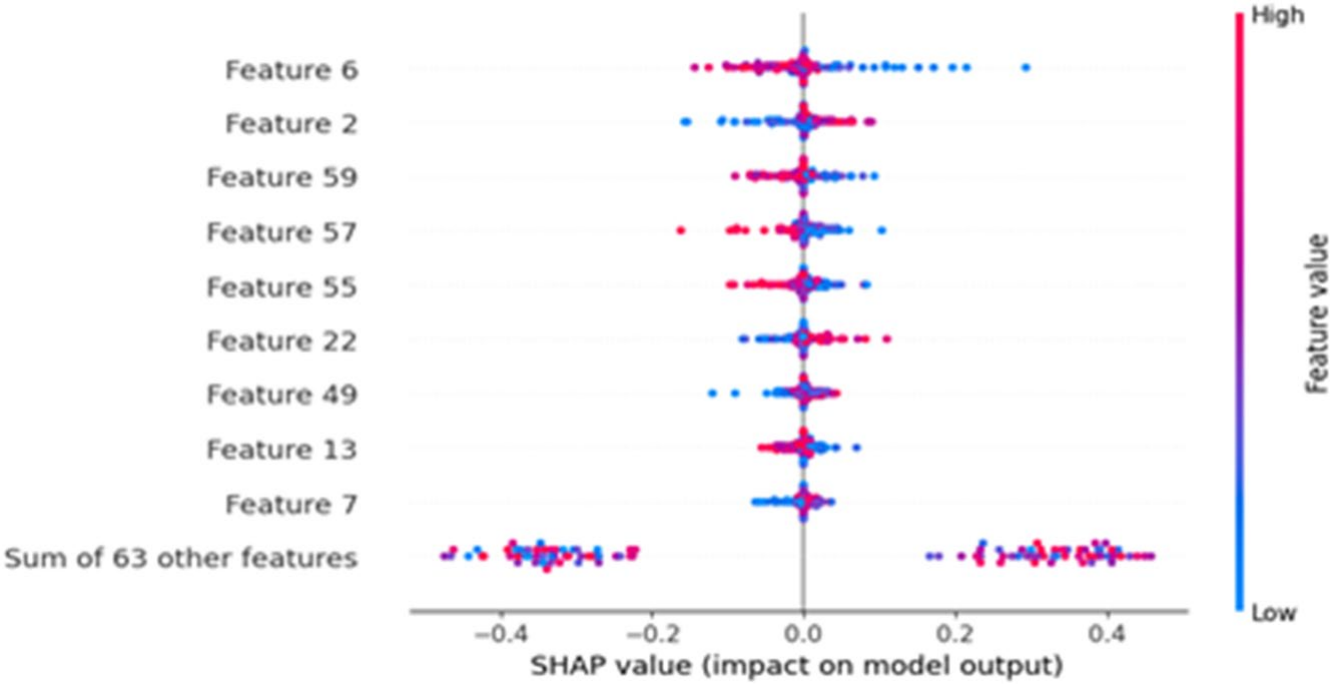
SHAP summary plot for extracted features using DWT for CHB-MIT dataset.

We have extracted DWT coefficients using the ‘db1’ mother wavelet and level 3, with signal length dependent on the data of each dataset. The extracted coefficients from DWT contain concatenated DWT coefficients which are: A3 (~ 0–16 Hz (delta-alpha)), D3 (~ 16–32 Hz (beta)), D2 (~ 32–64 Hz (gamma)), D1(~ 64–128 Hz (High gamma)). This feature extraction approach is repeated for each channel, and the results are then concatenated. The resulting data will be a sequence of 83-, 72-, or 4100-time steps (for different datasets), where each time step contains the wavelet coefficient for all channels at that DWT index. We have inputted a wavelet-transformed EEG tensor of shape (number of time points, 1), where each time point represents a specific wavelet coefficient across all EEG channels. SHAP value is used to explain the contribution of each feature in prediction. The SHAP summary plot of extracted features from DWT for CHB-MIT dataset is shown in Fig. 8. SHAP analysis highlighted feature 6 and feature 2, which originate from low-frequency (A3: 0–16 Hz) coefficients, which are highly influential, suggesting the role of delta/alpha-range activity in seizure prediction. Feature 59 from the gamma band (D2: 32–64 Hz) shows that the model is leveraging gamma-band abnormalities, which are known markers of seizure onset, to distinguish between seizure and non-seizure EEG patterns.

The model’s reliance on this feature 59 suggests it captures early seizure signatures in the gamma band, aligning with findings from clinical neurophysiology^53,54^.

Table 7 presents a comparative analysis of model complexity in terms of parameter count and floating-point operations (FLOPs) across our proposed method and three widely used EEG classification models. The proposed DWT-based 1D CNN-LSTM model has significantly fewer parameters than 2D CNN architectures, such as DeepConvNet and EEGNet, while maintaining competitive performance. Its FLOPs are lower across all evaluated datasets, demonstrating high computational efficiency. Unlike 2D CNNs, which rely on spatial convolutions over electrode topographies, our method achieves temporal-frequency representation via DWT and sequential modelling via LSTM, enabling efficient learning from both single-channel and multi-channel EEG data. This makes the proposed model especially suitable for resource-constrained or real-time applications.

**Table 7:** Comparison of model complexity with competitive EEG classification models.

Model	Architecture Type	# Parameters	FLOPs	Notes
Proposed model	**1D CNN-LSTM + DWT**	**0.35 M**	**1.67 × 10⁶ to 3.07 × 10⁷**	**Efficient; uses per-channel DWT and is suitable for both single/multi-channel EEG**
EEGNet^55^	2D CNN	0.5 M	~ 6 × 10⁷	Compact depthwise CNN; models spatial and spectral patterns
DeepConvNet^56^	2D CNN	1.7 M	~ 1 × 10⁸	Deeper architecture with high capacity and spatial modeling
CNN-LSTM^28^	1D CNN + LSTM	2.1 M	~ 5 × 10⁷	Models temporal dependencies; lacks explicit frequency-domain extraction

The best performances are in bold.

### Comparison with traditional and baseline methods

The performance of the proposed 1D CNN-LSTM model is compared with simpler baseline models, including a traditional CNN without LSTM and basic ML classifiers, to highlight its performance gains. Additionally, time-domain and frequency-domain analysis are used to assess the superiority of the proposed deep learning model over traditional EEG signal processing methods.

Time-domain analysis examines raw signal amplitude over time, extracting features like mean, standard deviation, and RMS to capture statistical and energy-related properties. These features help describe signal behavior, including variability and strength. Frequency-domain analysis, using Power Spectral Density (PSD) from Welch’s method, extracts feature like mean and median frequency to summarize the signal’s dominant frequency content. These features are vital in EEG analysis, as certain brain states or seizure patterns correlate with specific frequency bands. Comparison results for three benchmark datasets are shown in Tables 8 and 9.

**Table 8:** Comparison of the average accuracy of proposed model versus traditional methods for three datasets.

Datasets|methods	CNN	SVM	MLP	KNN	GNB	Proposed model
Bonn Epilepsy (binary)	91.23%	75.46%	87.5%	60%	89.34%	**98.02%**
CHB-MIT	70.90%	63.64%	40.1%	80%	52.72%	**96.94%**
TUSZ	84.27%	92.82%	92.74%	91.79%	79.15%	**94.32%**

The best performances are in bold.

**Table 9:** Comparison of the average accuracy of proposed model versus time-domain and frequency-domain analysis for three datasets.

Datasets|methods	Time-domain analysis	Frequency-domain analysis	Proposed model
Bonn epilepsy	66.74%	52.69%	**98.02%**
CHB-MIT	88.81%	82.83%	**96.94%**
TUSZ	84.15%	81.07%	**94.32%**

The best performances are in bold.

### Ablation studies

Ablation studies in deep learning are experimental methods to evaluate the importance of particular components or modules within a model by removing or modifying them and observing their impact on the model’s performance^57^. The term “ablation” originates from the biological practice of surgically removing tissues or organs to study their function, particularly in the context of ablative brain surgery in neuropsychology, where certain brain areas are removed from animals to analyze behavioral changes. This analogy extends to deep learning, where parts of the network are removed to study the resulting behavior and changes in performance. In this section, we present ablation studies to evaluate the impact of various components of the proposed model on its performance. The results of ablation studies for three datasets are given in Table 10.

The results in Table 10 indicate that the CHB-MIT dataset exhibits a substantially larger performance drop when individual components of the proposed model—DWT, LSTM, or CNN—are removed. Specifically, removing DWT, LSTM, or CNN results in significant reductions in accuracy (from 96.94 to 69.05%, 64.37%, and 76.82%, respectively), whereas the Bonn and TUSZ datasets exhibit more moderate variations. This discrepancy can be attributed to the inherent complexity and variability of the CHB-MIT dataset, which contains long-term, real-world EEG recordings from pediatric patients with diverse seizure types and conditions. Unlike the Bonn dataset, which is well-segmented and clean, and the TUSZ dataset, which is partially curated and labeled, CHB-MIT is noisier and less homogeneous. As a result, effective feature extraction (via DWT), temporal modeling (via LSTM), and spatial feature extraction (via CNN) all play critical roles in achieving high performance on CHB-MIT.

In contrast, the Bonn dataset consists of short, artifact-free EEG segments, where even simpler models can perform well. Similarly, while TUSZ is complex, it may benefit more from temporal modeling (LSTM) than from frequency decomposition (DWT), as indicated by the relatively small drop in performance when DWT is removed. These findings imply that model components contribute differently depending on dataset characteristics. For complex, real-world datasets like CHB-MIT, the synergy of all model components is crucial for effectively capturing both temporal and frequency dynamics.

The proposed hybrid model leverages the complementary strengths of DWT and CNN-LSTM architectures. DWT decomposes EEG signals into multi-resolution components, effectively capturing both transient and sustained frequency features across different scales. By isolating the pertinent frequency bands associated with seizure activity, this frequency-domain decomposition enhances the separability of features. The CNN layers then learn spatially localized patterns within these frequency-decomposed signals, so extracting salient features across time-frequency representations. Ultimately, the LSTM layers facilitate the capture of natural rhythms and patterns that emerge over time in EEG signals, thereby facilitating the recognition of key temporal trends. By combining the multi-resolution analysis of DWT with the powerful spatial and temporal modelling capabilities of the CNN-LSTM, this approach yields more effective feature extraction and improved classification results compared to using a CNN or LSTM alone.

**Table 10:** Comparison of the average accuracy of proposed model versus model without DWT and model without LSTM.

Datasets|ablation methods	Without DWT	Without LSTM	Without CNN	Proposed model (with DWT and with LSTM)
Bonn Epilepsy	97.49%	98.01%	80.23%	**98.02%**
CHB-MIT	69.05%	64.37%	76.82%	**96.94%**
TUSZ	88.9%	91.21%	87.52%	**94.32%**

The best performances are in bold.

## Discussion

Working with real-world EEG data is always challenging. For example, real-world data is more noisy than publicly available datasets. Sometimes, EEG is contaminated by other bio-signals, such as EOG, EMG, or ECG signals. Therefore, it is essential to utilize robust and reliable noise reduction mechanisms with the proposed model when working with real-world data, especially when processing data in real time, given the sensitivity of working with EEG data from individuals with epilepsy. The most important finding of this work is that for any type of epilepsy seizure EEG signal (whether binary or multi-class classification or whether one-channel or multi-channel EEG signal), the 1D CNN-LSTM model with concatenated DWT features can lead to salient outputs. The proposed model utilizes a 1D CNN, which has fewer parameters than a 2D CNN, and combines both CNN and LSTM networks to provide a comprehensive view of EEG signals from two different perspectives, thereby extracting diverse features from them. During the model training and design, several limitations arise. Training deep learning models requires a significant amount of computing power and memory, which can be challenging to access. That is why we opted for Google Colab Pro, as the free version does not provide sufficient resources for practical training.

Additionally, applying the model to entirely new datasets bring its unique challenges. Differences between individuals, unique dataset characteristics, and variability across subjects all affect how well the model performs. To tackle this, real-time evaluation and feedback from both patients and clinicians can be constructive. Future research could focus on testing and refining this model in real-time clinical settings. Future work may explore the incorporation of spatial relationships more explicitly, for example, through convolutional layers with spatial kernels or graph-based models that leverage electrode layout information. One promising direction for future research involves testing the proposed model’s performance in real-time on edge devices. It will be crucial to assess how effectively the model strikes a balance between inference speed and memory consumption, as well as its ease of deployment on hardware such as the NVIDIA Jetson Nano or Coral TPU. These evaluations are essential for understanding whether the model is truly viable for time-critical clinical settings and practical use in portable seizure alert systems.

Although the DWT provides an effective time-frequency decomposition for EEG analysis, it suffers from the shift-variant problem. This means that small temporal shifts in the input signal can lead to significant alterations in the wavelet coefficients and reduce the feature stability and robustness. Researchers in future work can investigate shift-invariant versions such as the complex wavelet transform (CWT) and the dual-tree complex wavelet transform (DT-CWT). The main task of these methods is to offer approximate shift invariance^58,59^Such enhancements will improve the robustness of EEG feature extraction.

While our model demonstrates promising performance across multiple datasets, translating such technology into clinical practice requires careful consideration of ethical, safety, and regulatory factors. Medical deployment of EEG-based seizure detection systems must comply with relevant standards and regulations, such as FDA clearance in the United States, CE marking in Europe, and adherence to IEC 80601-2-77, which governs the safety and essential performance of electroencephalographic equipment. Additionally, stringent data privacy and security measures must be in place when handling EEG data in real-world settings by laws such as HIPAA or GDPR. While this study utilized de-identified public datasets, future clinical applications must prioritize patient confidentiality by implementing secure data storage, encryption, and access controls. Meeting these requirements is crucial for the safe, effective, and ethically responsible integration of AI-based diagnostic tools in healthcare settings.

## Conclusion and future works

The novel neural network-based architecture aims to extract both spatial and temporal features from EEG signals, leveraging both CNN and LSTM networks. The 1D CNN plays a crucial role in the proposed model. By extracting local and general features from its input (DWT features), 1D CNN helps to superior model performance. The proposed model has been evaluated on three publicly available datasets in comparison with other traditional machine learning classifiers, such as KNN and SVC. Deep learning models have demonstrated significant applicability in various fields. Without extensive preprocessing steps, these models can be utilized as detection systems for EEG signals. Minimal preprocessing was applied to the EEG data. First, the continuous EEG recordings were segmented into fixed-length time windows to structure the input for the model. Following segmentation, each window was normalized to have zero mean and unit variance.

The most challenging part of designing these networks is their architecture. Another challenge is the lack of suitable recorded EEG data, which is time-consuming and expensive. In future work, researchers can investigate data augmentation in deep learning models to generate more informative and discriminative artificial EEG data, thereby training models more effectively. Also, future works can assess the application of other state-of-the-art deep learning models in epileptic EEG data detection. These models are even developed for image, text, or audio. Their applicability for EEG signal processing can be a new research topic.

## Supplementary Information

Below is the link to the electronic supplementary material.

Supplementary Material 1

## Data Availability

The data analyzed in this study were a re-examination of publicly available datasets, which are described and properly referenced in the manuscript. Additionally, the complete code for the experiments, including dataset preprocessing and evaluation, can be accessed at [https://github.com/Homakashefi/1D-CNN-LSTM-for-Seizure-Detection-EEG-Signals-/tree/main](https:/github.com/Homakashefi/1D-CNN-LSTM-for-Seizure-Detection-EEG-Signals-/tree/main) .
