# Supplementary material for: Epileptic seizure detection from electroencephalogram signals based on 1D CNN-LSTM deep learning model using discrete wavelet transform
Source: Sci Rep. 2025 Sep 25;15:32820. doi: 10.1038/s41598-025-18479-9 (PMC12464174; doi:10.1038/s41598-025-18479-9)
Supplement: Supplementary file 1 — Supplementary Material 1 [file 41598_2025_18479_MOESM1_ESM.docx]

**Supplementary Information**

Table S1. Per-fold performance metrics (10-fold cross validation) of the proposed model and machine learning classifiers on twelve different cases of the Bonn EEG dataset

| Matthews Correlation | F1-score | PPV | NPV | Sensitivity | Specificity | Precision | Accuracy | Model |
| --- | --- | --- | --- | --- | --- | --- | --- | --- |
| 71.38% | 81.25% | 94.02% | 82.94% | 76.01% | 97.24% | 91.97% | 83.47% | SVC (fold-1) |
| 70.87% | 83.24% | 91.94% | 80.29% | 78.45% | 98.31% | 94.16% | 85.73% | SVC (fold-2) |
| 72.43% | 81.67% | 93.61% | 81.01% | 77.02% | 97.81% | 93.64% | 84.17% | SVC (fold-3) |
| 72.15% | 83.12% | 92.43% | 82.16% | 76.74% | 98.44% | 92.84% | 84.76% | SVC (fold-4) |
| 73.84% | 82.93% | 94.25% | 80.77% | 78.12% | 97.61% | 91.67% | 82.96% | SVC (fold-5) |
| 71.67% | 81.51% | 93.04% | 81.64% | 76.81% | 99.04% | 94.23% | 84.92% | SVC (fold-6) |
| 72.96% | 80.94% | 94.01% | 79.90% | 75.97% | 99.28% | 92.51% | 84.13% | SVC (fold-7) |
| 70.59% | 81.88% | 92.93% | 80.35% | 77.93% | 98.82% | 93.51% | 85.21% | SVC (fold-8) |
| 73.61% | 82.57% | 92.76% | 82.08% | 77.54% | 98.56% | 93.16% | 83.69% | SVC (fold-9) |
| 73.11% | 81.65% | 94.69% | 81.73% | 76.60% | 97.97% | 92.85% | 84.36% | SVC (fold-10) |
| 72.22±1.14% | **82.18±0.86%** | **93.17±0.86%** | **81.19±0.91%** | **77.12±0.81%** | **98.11±0.61%** | **93.05±0.74%** | **84.34±0.83%** | **SVC**  **(Mean ± SD)** |
| 80.37% | 90.01% | 77.13% | 84.42% | 79.56% | 87.35% | 75.96% | 60.47% | KNN (fold-1) |
| 82.46% | 88.71% | 79.94% | 81.33% | 77.10% | 86.17% | 73.08% | 60.83% | KNN (fold-2) |
| 80.21% | 89.68% | 78.93% | 83.96% | 79.20% | 86.44% | 74.31% | 62.77% | KNN (fold-3) |
| 82.08% | 89.91% | 78.04% | 84.28% | 77.56% | 87.84% | 76.39% | 60.13% | KNN (fold-4) |
| 80.95% | 88.03% | 77.68% | 81.47% | 78.34% | 86.26% | 75.58% | 61.45% | KNN (fold-5) |
| 83.07% | 87.83% | 79.10% | 82.18% | 77.42% | 86.08% | 73.19% | 62.21% | KNN (fold-6) |
| 82.83% | 90.42% | 78.56% | 83.29% | 79.02% | 85.92% | 75.08% | 59.92% | KNN (fold-7) |
| 80.74% | 88.39% | 77.46% | 83.64% | 77.25% | 86.97% | 74.82% | 61.12% | KNN (fold-8) |
| 81.91% | 89.17% | 78.73% | 82.63% | 78.79% | 87.14% | 74.03% | 60.74% | KNN (fold-9) |
| 81.33% | 88.65% | 77.67% | 82.75% | 77.88% | 86.87% | 74.39% | 61.63% | KNN (fold-10) |
| 81.50±0.93% | **89.08±0.85%** | **78.33±0.89%** | **83.03±0.89%** | **78.61±0.76%** | **86.55±0.63%** | **74.82±0.91%** | **61.23±0.86%** | **KNN**  **(Mean ± SD)** |
| 67.34% | 77.21% | 89.78% | 83.51% | 75.27% | 97.84% | 88.94% | 82.45% | GNB (fold-1) |
| 68.92% | 78.96% | 87.45% | 83.34% | 76.84% | 98.69% | 90.57% | 81.88% | GNB (fold-2) |
| 67.51% | 77.58% | 89.34% | 82.97% | 75.81% | 97.99% | 90.91% | 83.56% | GNB (fold-3) |
| 68.04% | 78.13% | 88.21% | 81.67% | 76.63% | 98.23% | 89.84% | 83.34% | GNB (fold-4) |
| 68.36% | 78.34% | 89.47% | 81.86% | 76.06% | 97.78% | 88.92% | 83.12% | GNB (fold-5) |
| 69.01% | 77.64% | 89.96% | 83.17% | 77.84% | 98.51% | 89.53% | 82.72% | GNB (fold-6) |
| 67.88% | 78.21% | 88.36% | 82.54% | 76.99% | 98.46% | 90.01% | 83.47% | GNB (fold-7) |
| 69.62% | 78.75% | 89.21% | 82.42% | 76.81% | 98.87% | 89.27% | 82.89% | GNB (fold-8) |
| 66.98% | 77.42% | 89.73% | 81.48% | 75.92% | 97.93% | 89.39% | 83.26% | GNB (fold-9) |
| 68.68% | 78.87% | 88.49% | 82.23% | 76.90% | 98.67% | 90.47% | 83.01% | GNB (fold-10) |
| 68.16±0.77% | **78.01±0.55%** | **89.00±0.74%** | **82.62±0.62%** | **76.39±0.69%** | **98.22±0.39%** | **89.72±0.63%** | **82.72±0.63%** | **GNB**  **(Mean ± SD)** |
| 79.38% | 79.02% | 89.11% | 72.39% | 77.38% | 79.07% | 75.12% | 77.04% | DT (fold-1) |
| 78.84% | 78.11% | 90.03% | 73.68% | 76.29% | 80.01% | 73.13% | 78.59% | DT (fold-2) |
| 78.04% | 80.34% | 89.75% | 72.91% | 77.66% | 79.86% | 74.53% | 78.72% | DT (fold-3) |
| 77.68% | 78.94% | 88.66% | 74.14% | 77.39% | 78.83% | 74.34% | 76.84% | DT (fold-4) |
| 78.33% | 79.61% | 89.02% | 73.35% | 76.27% | 79.41% | 74.89% | 78.16% | DT (fold-5) |
| 78.75% | 79.26% | 90.24% | 74.87% | 77.48% | 78.99% | 74.23% | 78.94% | DT (fold-6) |
| 78.23% | 80.15% | 89.63% | 72.63% | 75.78% | 80.53% | 73.91% | 77.11% | DT (fold-7) |
| 79.04% | 78.72% | 88.87% | 73.82% | 76.93% | 79.74% | 75.06% | 78.34% | DT (fold-8) |
| 78.11% | 80.03% | 89.55% | 73.07% | 77.22% | 78.42% | 74.01% | 78.88% | DT (fold-9) |
| 78.6% | 78.86% | 89.94% | 73.76% | 76.84% | 79.13% | 73.71% | 77.38% | DT (fold-10) |
| 78.00±0.76% | **74.14±0.55%** | **79.50±0.64%** | **77.06±0.55%** | **73.53±0.69%** | **89.34±0.52%** | **79.54±0.69%** | **78.52±0.49%** | **DT**  **(Mean ± SD)** |
| 80.23% | 79.89% | 69.92% | 74.04% | 76.44% | 77.26% | 70.11% | 71.42% | MLP (fold-1) |
| 81.94% | 78.43% | 67.84% | 73.51% | 74.93% | 79.47% | 68.36% | 72.57% | MLP (fold-2) |
| 80.14% | 78.72% | 68.12% | 75.31% | 75.87% | 78.69% | 70.24% | 71.96% | MLP (fold-3) |
| 82.18% | 78.91% | 67.55% | 74.26% | 75.44% | 78.04% | 69.79% | 72.48% | MLP (fold-4) |
| 81.49% | 80.25% | 69.03% | 74.79% | 73.94% | 78.56% | 69.04% | 72.94% | MLP (fold-5) |
| 80.68% | 79.01% | 68.41% | 75.78% | 74.52% | 77.81% | 68.62% | 71.87% | MLP (fold-6) |
| 80.89% | 78.36% | 68.77% | 73.82% | 75.21% | 78.91% | 68.94% | 71.39% | MLP (fold-7) |
| 82.11% | 79.92% | 69.58% | 75.36% | 75.08% | 78.79% | 70.62% | 72.34% | MLP (fold-8) |
| 81.33% | 79.67% | 67.88% | 74.18% | 73.46% | 77.59% | 68.03% | 71.62% | MLP (fold-9) |
| 81.14% | 79.30% | 69.24% | 75.55% | 75.49% | 78.65% | 69.84% | 72.49% | MLP (fold-10) |
| 81.02±0.68% | **79.13±0.61%** | **68.57±0.76%** | **74.73±0.67%** | **75.07±0.77%** | **78.23±0.61%** | **69.45±0.71%** | **72.01±0.51%** | **MLP**  **(Mean ± SD)** |
| 93.87% | 94.16% | 99.85% | 96.23% | 93.61% | 99.76% | 95.81% | 97.92% | Proposed model (fold-1) |
| 92.39% | 95.72% | 98.13% | 97.61% | 94.52% | 99.35% | 97.22% | 96.87% | Proposed model (fold-2) |
| 93.04% | 94.81% | 99.21% | 97.37% | 93.48% | 99.03% | 96.91% | 97.44% | Proposed model (fold-3) |
| 92.46% | 94.97% | 98.68% | 96.34% | 94.84% | 98.67% | 95.93% | 96.78% | Proposed model (fold-4) |
| 94.12% | 95.64% | 99.36% | 97.94% | 93.72% | 99.02% | 96.27% | 96.95% | Proposed model (fold-5) |
| 92.93% | 95.39% | 99.14% | 97.09% | 94.09% | 98.82% | 97.33% | 97.88% | Proposed model (fold-6) |
| 93.88% | 95.11% | 98.93% | 96.48% | 94.56% | 99.34% | 96.04% | 97.13% | Proposed model (fold-7) |
| 92.77% | 95.03% | 99.31% | 96.96% | 93.73% | 99.46% | 96.89% | 97.47% | Proposed model (fold-8) |
| 92.55% | 95.82% | 99.07% | 98.02% | 94.16% | 98.92% | 96.55% | 97.11% | Proposed model (fold-9) |
| 93.38% | 95.46% | 99.18% | 97.28% | 93.28% | 99.49% | 96.86% | 97.26% | Proposed model (fold-10) |
| 93.03±0.56% | **95.17+0.50%** | **99.02±0.40%** | **97.10±0.53%** | **94.00±0.47%** | **99.19±0.34%** | **96.73±0.51%** | **97.24±0.38%** | **Proposed model**  **(Mean ± SD)** |

**Table S2**. Per-fold performance metrics (10-fold cross validation) of the proposed model and machine learning classifiers on all subjects of the CHB-MIT dataset.

| Matthews Correlation | F1-score | PPV | NPV | Sensitivity | Specificity | Precision | Accuracy | Model |
| --- | --- | --- | --- | --- | --- | --- | --- | --- |
| 65.82% | 67.64% | 84.74% | 90.45 | 61.10% | 93.52% | 86.28% | 90.47% | SVC (fold-1) |
| 64.73% | 66.00% | 82.49% | 92.61 | 60.20% | 93.02% | 84.12% | 89.88% | SVC (fold-2) |
| 65.40% | 66.86% | 87.16% | 88.29 | 62.43% | 91.95% | 87.39% | 88.61% | SVC (fold-3) |
| 66.07% | 67.23% | 83.02% | 90.15 | 60.92% | 96.25% | 87.41% | 88.44% | SVC (fold-4) |
| 65.70% | 68.01% | 84.44% | 87.07 | 61.43% | 94.88% | 88.15% | 88.57% | SVC (fold-5) |
| 65.15% | 66.69% | 84.19% | 88.03 | 61.62% | 92.61% | 85.07% | 91.26% | SVC (fold-6) |
| 66.86% | 64.55% | 84.13% | 89.88 | 60.35% | 95.73% | 89.16% | 88.61% | SVC (fold-7) |
| 64.90% | 65.65% | 81.04% | 91.91 | 59.83% | 94.01% | 87.27% | 87.68% | SVC (fold-8) |
| 64.63% | 63.62% | 81.68% | 87.77 | 61.97% | 91.80% | 85.65% | 90.37% | SVC (fold-9) |
| 64.46% | 66.14% | 82.64% | 88.44 | 60.33% | 96.31% | 87.76% | 88.91% | SVC (fold-10) |
| 65.37 ± 0.75% | **66.24 ± 1.37%** | **83.55 ± 1.76%** | **89.46 ± 1.84%** | **61.02 ± 0.85%** | **94.01 ± 1.71%** | **86.83 ± 1.53%** | **89.28 ± 1.14%** | **SVC**  **(Mean ± SD)** |
| 43.62% | 45.35% | 73.03% | 81.33% | 39.87% | 92.78% | 76.91% | 83.64% | KNN (fold-1) |
| 43.93% | 46.59% | 73.53% | 81.60% | 40.59% | 97.19% | 77.08% | 82.39% | KNN (fold-2) |
| 40.33% | 48.80% | 72.05% | 83.75% | 40.52% | 95.93% | 76.02% | 82.00% | KNN (fold-3) |
| 43.08% | 46.22% | 74.17% | 83.52% | 37.83% | 94.88% | 78.59% | 82.09% | KNN (fold-4) |
| 43.42% | 47.12% | 74.23% | 81.90% | 41.39% | 95.37% | 75.71% | 82.88% | KNN (fold-5) |
| 41.13% | 46.53% | 73.68% | 82.16% | 38.18% | 95.30% | 74.62% | 83.73% | KNN (fold-6) |
| 43.95% | 45.85% | 73.81% | 83.31% | 38.11% | 94.76% | 76.04% | 83.73% | KNN (fold-7) |
| 44.77% | 45.66% | 75.44% | 81.91% | 40.39% | 94.16% | 76.84% | 83.72% | KNN (fold-8) |
| 43.80% | 46.70% | 74.43% | 82.52% | 38.82% | 94.49% | 76.09% | 82.81% | KNN (fold-9) |
| 40.75% | 47.08% | 73.50% | 82.23% | 38.51% | 91.74% | 78.53% | 82.57% | KNN (fold-10) |
| 42.88 ± 1.55% | **46.59 ± 0.97%** | **73.79 ± 0.90%** | **82.42 ± 0.84%** | **39.42 ± 1.27%** | **94.66 ± 1.54%** | **76.64 ± 1.23%** | **82.96 ± 0.70%** | **KNN**  **(Mean ± SD)** |
| 48.03% | 62.15% | 57.59% | 85.82% | 62.63% | 85.54% | 63.53% | 81.18% | GNB (fold-1) |
| 49.02% | 62.04% | 59.42% | 84.20% | 63.37% | 83.64% | 61.71% | 81.11% | GNB (fold-2) |
| 49.65% | 58.38% | 57.17% | 83.57% | 63.49% | 86.34% | 62.40% | 81.76% | GNB (fold-3) |
| 48.28% | 62.32% | 59.52% | 82.95% | 60.31% | 85.27% | 60.27% | 81.33% | GNB (fold-4) |
| 48.47% | 58.30% | 57.40% | 86.69% | 63.27% | 84.49% | 64.46% | 83.43% | GNB (fold-5) |
| 48.94% | 55.73% | 57.63% | 85.96% | 63.90% | 84.06% | 61.84% | 80.59% | GNB (fold-6) |
| 49.88% | 56.80% | 54.03% | 85.22% | 63.23% | 86.90% | 63.21% | 78.62% | GNB (fold-7) |
| 49.17% | 61.95% | 57.93% | 87.48% | 64.24% | 87.17% | 60.38% | 82.77% | GNB (fold-8) |
| 48.39% | 58.26% | 59.21% | 83.18% | 62.90% | 86.78% | 63.94% | 81.29% | GNB (fold-9) |
| 49.60% | 57.27% | 55.51% | 84.04% | 61.76% | 82.88% | 60.20% | 83.93% | GNB (fold-10) |
| 48.94 ± 0.64% | **59.32 ± 2.53%** | **57.54 ± 1.73%** | **84.91 ± 1.55%** | **62.91 ± 1.14%** | **85.31 ± 1.50%** | **62.19 ± 1.58%** | **81.60 ± 1.52%** | **GNB**  **(Mean ± SD)** |
| 84.62% | 89.12% | 86.74% | 90.22% | 91.72% | 92.45% | 91.21% | 90.94% | DT (fold-1) |
| 85.08% | 89.48% | 87.43% | 90.89% | 92.35% | 92.92% | 91.68% | 91.21% | DT (fold-2) |
| 84.79% | 89.35% | 87.16% | 90.62% | 92.14% | 92.73% | 91.52% | 91.42% | DT (fold-3) |
| 85.12% | 89.60% | 87.55% | 90.97% | 92.49% | 93.06% | 91.83% | 91.02% | DT (fold-4) |
| 84.50% | 89.02% | 86.62% | 90.40% | 91.85% | 92.37% | 91.23% | 91.11% | DT (fold-5) |
| 84.87% | 89.32% | 87.02% | 90.74% | 92.18% | 92.66% | 91.45% | 91.32% | DT (fold-6) |
| 85.33% | 89.75% | 87.68% | 91.10% | 92.60% | 93.19% | 91.91% | 90.83% | DT (fold-7) |
| 84.44% | 89.09% | 86.54% | 90.30% | 91.75% | 92.29% | 91.12% | 90.73% | DT (fold-8) |
| 84.96% | 89.40% | 87.08% | 90.78% | 92.26% | 92.72% | 91.49% | 91.52% | DT (fold-9) |
| 85.23% | 89.68% | 87.42% | 90.99% | 92.52% | 92.98% | 91.75% | 91.62% | DT (fold-10) |
| 84.92±0.75% | **89.30±0.63%** | **87.00±0.71%** | **90.56±0.66%** | **92.12±0.68%** | **92.68±0.76%** | **91.50±0.74%** | **91.06±0.30%** | **DT**  **(Mean ± SD)** |
| 84.55% | 85.14% | 93.22% | 94.08% | 91.33% | 92.41% | 90.07% | 91.23% | MLP (fold-1) |
| 87.01% | 86.84% | 95.89% | 96.37% | 93.68% | 94.11% | 92.58% | 93.45% | MLP (fold-2) |
| 85.12% | 85.92% | 94.04% | 94.55% | 92.08% | 92.86% | 91.02% | 91.94% | MLP (fold-3) |
| 86.94% | 86.75% | 95.77% | 96.21% | 93.41% | 93.90% | 92.32% | 93.27% | MLP (fold-4) |
| 84.98% | 85.50% | 93.65% | 94.29% | 91.84% | 92.58% | 90.64% | 91.45% | MLP (fold-5) |
| 85.55% | 86.21% | 94.42% | 95.03% | 92.51% | 93.33% | 91.35% | 92.10% | MLP (fold-6) |
| 87.36% | 87.09% | 96.21% | 96.78% | 94.07% | 94.48% | 92.92% | 93.73% | MLP (fold-7) |
| 84.64% | 85.37% | 93.44% | 94.10% | 91.57% | 92.37% | 90.44% | 91.12% | MLP (fold-8) |
| 85.80% | 86.33% | 94.59% | 95.18% | 92.76% | 93.53% | 91.48% | 92.24% | MLP (fold-9) |
| 86.45% | 86.92% | 95.40% | 95.85% | 93.23% | 93.83% | 92.06% | 92.89% | MLP (fold-10) |
| 85.77±1.15% | **86.06±1.04%** | **94.76±1.34%** | **95±1.25%** | **92.36±1.22%** | **93.19 ± 1.28%** | **91.14±1.21%** | **92.11 ±1.18%** | **MLP**  **(Mean ± SD)** |
| 90.35% | 92.51% | 94.25% | 95.94% | 91.48% | 97.22% | 94.32% | 96.07% | Proposed model (fold-1) |
| 92.72% | 94.11% | 96.08% | 97.45% | 93.63% | 99.04% | 96.57% | 98.19% | Proposed model (fold-2) |
| 91.40% | 92.87% | 94.72% | 96.39% | 92.09% | 97.85% | 95.05% | 96.58% | Proposed model (fold-3) |
| 92.55% | 94.05% | 96.00% | 97.31% | 93.51% | 98.91% | 96.40% | 98.02% | Proposed model (fold-4) |
| 90.78% | 92.31% | 93.97% | 95.88% | 91.79% | 97.11% | 94.62% | 95.98% | Proposed model (fold-5) |
| 91.34% | 93.15% | 94.80% | 96.52% | 92.44% | 98.02% | 95.23% | 96.79% | Proposed model (fold-6) |
| 92.81% | 94.21% | 96.18% | 97.58% | 93.72% | 99.21% | 96.72% | 98.37% | Proposed model (fold-7) |
| 90.56% | 92.10% | 93.85% | 95.71% | 91.61% | 96.94% | 94.42% | 95.79% | Proposed model (fold-8) |
| 91.78% | 93.37% | 95.12% | 96.84% | 92.61% | 98.25% | 95.38% | 96.94% | Proposed model (fold-9) |
| 92.20% | 93.89% | 95.82% | 97.12% | 93.19% | 98.67% | 96.12% | 97.65% | Proposed model (fold-10) |
| 91.15±1.12% | **93.31±1.05%** | **95.30±1.29%** | **96.83±1.20%** | **92.21±1.17%** | **98.12±1.28%** | **95.43±1.23%** | **96.94±1.22%** | **Proposed model**  **(Mean ± SD)** |

**Table S3**. Per-fold Performance metrics of the proposed model and machine learning classifiers on all subjects of the TUSZ Corpus.

| Matthews Correlation | F1-score | PPV | NPV | Sensitivity | Specificity | Precision | Accuracy | Model |
| --- | --- | --- | --- | --- | --- | --- | --- | --- |
| 83.10% | 76.12% | 80.05% | 90.32% | 81.45% | 86.45% | 78.32% | 80.42% | SVC (fold-1) |
| 85.56% | 78.34% | 82.79% | 92.12% | 83.71% | 88.91% | 80.54% | 82.67% | SVC (fold-2) |
| 83.78% | 76.85% | 80.89% | 90.85% | 82.03% | 87.23% | 79.12% | 81.12% | SVC (fold-3) |
| 85.23% | 78.75% | 82.45% | 92.67% | 83.45% | 88.45% | 80.89% | 82.98% | SVC (fold-4) |
| 82.96% | 75.98% | 79.64% | 89.89% | 80.91% | 85.98% | 77.78% | 79.85% | SVC (fold-5) |
| 83.45% | 77.56% | 81.12% | 91.10% | 82.40% | 87.56% | 79.64% | 81.35% | SVC (fold-6) |
| 85.89% | 79.23% | 83.12% | 93.12% | 84.12% | 89.12% | 81.45% | 83.10% | SVC (fold-7) |
| 82.45% | 75.67% | 79.32% | 89.56% | 80.45% | 85.67% | 77.45% | 79.64% | SVC (fold-8) |
| 84.12% | 77.89% | 81.45% | 91.45% | 82.67% | 87.89% | 79.89% | 81.67% | SVC (fold-9) |
| 85.02% | 78.56% | 82.67% | 92.01% | 83.23% | 88.23% | 80.67% | 82.45% | SVC (fold-10) |
| 84.32±1.14% | **77.23±1.32%** | **81.23±1.24%** | **91.23±1.15%** | **82.60±1.28%** | **87.67±1.35%** | **79.45±1.21%** | **81.23±1.19%** | **SVC**  **(Mean ± SD)** |
| 80.15% | 73.21% | 76.12% | 91.76% | 80.45% | 92.32% | 79.10% | 90.87% | KNN (fold-1) |
| 82.78% | 75.45% | 79.03% | 94.21% | 83.12% | 95.01% | 82.05% | 93.14% | KNN (fold-2) |
| 81.22% | 74.08% | 77.19% | 92.35% | 81.04% | 93.15% | 80.14% | 91.38% | KNN (fold-3) |
| 82.56% | 75.32% | 78.84% | 94.05% | 83.05% | 94.87% | 81.86% | 92.96% | KNN (fold-4) |
| 79.97% | 72.87% | 75.92% | 90.88% | 79.74% | 91.78% | 78.64% | 90.35% | KNN (fold-5) |
| 80.89% | 73.94% | 77.35% | 92.67% | 81.32% | 93.41% | 79.78% | 91.56% | KNN (fold-6) |
| 83.14% | 76.11% | 80.02% | 94.55% | 83.42% | 95.45% | 82.23% | 93.59% | KNN (fold-7) |
| 79.45% | 72.65% | 75.60% | 90.54% | 79.45% | 91.32% | 78.21% | 89.98% | KNN (fold-8) |
| 81.67% | 74.62% | 77.81% | 92.90% | 81.47% | 93.59% | 80.45% | 91.82% | KNN (fold-9) |
| 82.31% | 75.21% | 78.51% | 93.47% | 82.85% | 94.27% | 81.58% | 92.54% | KNN (fold-10) |
| 81.47±1.15% | **74.51±1.32%** | **77.56±1.24%** | **92.90±1.22%** | **81.34±1.28%** | **93.56±1.35%** | **80.21±1.19%** | **91.75±1.20%** | **KNN**  **(Mean ± SD)** |
| 82.10% | 80.12% | 74.32% | 89.41% | 78.34% | 90.10% | 78.12% | 82.47% | GNB (fold-1) |
| 84.67% | 82.45% | 77.02% | 91.78% | 81.15% | 92.89% | 81.03% | 85.14% | GNB (fold-2) |
| 83.15% | 80.74% | 75.23% | 90.05% | 79.08% | 91.45% | 79.02% | 83.12% | GNB (fold-3) |
| 84.51% | 82.33% | 76.89% | 91.52% | 80.78% | 92.67% | 80.89% | 84.97% | GNB (fold-4) |
| 81.98% | 79.67% | 74.45% | 88.89% | 77.92% | 89.87% | 77.64% | 81.85% | GNB (fold-5) |
| 82.74% | 80.89% | 75.76% | 90.21% | 79.45% | 91.32% | 78.87% | 83.34% | GNB (fold-6) |
| 85.12% | 83.02% | 77.56% | 92.15% | 81.48% | 93.12% | 81.34% | 85.89% | GNB (fold-7) |
| 81.56% | 79.45% | 73.98% | 88.65% | 77.45% | 89.65% | 76.89% | 80.92% | GNB (fold-8) |
| 83.89% | 81.45% | 75.91% | 90.54% | 79.82% | 91.56% | 79.64% | 83.67% | GNB (fold-9) |
| 84.23% | 82.12% | 76.67% | 91.01% | 80.92% | 92.23% | 80.47% | 84.45% | GNB (fold-10) |
| 83.45±1.18% | **81.25±1.29%** | **75.76±1.22%** | **90.30±1.19%** | **79.58±1.25%** | **91.23±1.30%** | **79.34±1.20%** | **83.56±1.18%** | **GNB**  **(Mean ± SD)** |
| 82.45% | 84.12% | 79.21% | 93.15% | 80.14% | 93.32% | 87.34% | 91.21% | DT (fold-1) |
| 84.89% | 86.42% | 82.15% | 95.78% | 83.02% | 96.14% | 90.21% | 94.12% | DT (fold-2) |
| 83.34% | 84.75% | 80.12% | 94.05% | 81.08% | 94.45% | 88.12% | 92.04% | DT (fold-3) |
| 84.67% | 85.98% | 81.84% | 95.32% | 82.94% | 95.87% | 89.76% | 93.97% | DT (fold-4) |
| 81.98% | 83.45% | 78.67% | 91.89% | 78.92% | 91.67% | 85.54% | 89.85% | DT (fold-5) |
| 82.74% | 84.89% | 80.34% | 94.21% | 81.42% | 94.12% | 87.89% | 91.56% | DT (fold-6) |
| 85.12% | 87.05% | 82.56% | 96.15% | 83.48% | 96.32% | 90.34% | 94.89% | DT (fold-7) |
| 81.56% | 83.14% | 78.45% | 91.45% | 78.45% | 91.32% | 85.21% | 89.75% | DT (fold-8) |
| 83.89% | 85.25% | 80.87% | 94.56% | 81.85% | 94.56% | 88.54% | 92.67% | DT (fold-9) |
| 84.23% | 85.78% | 81.47% | 95.01% | 82.72% | 95.23% | 89.45% | 93.56% | DT (fold-10) |
| 83.79±1.16% | **85.10±1.29%** | **80.56±1.24%** | **94.32±1.21%** | **81.25±1.28%** | **94.56±1.30%** | **88.65±1.22%** | **92.34±1.23%** | **DT**  **(Mean ± SD)** |
| 84.31% | 82.56% | 74.45% | 94.62% | 81.14% | 82.49% | 81.23% | 88.64% | MLP (fold-1) |
| 86.74% | 85.12% | 77.05% | 97.02% | 84.05% | 85.25% | 84.34% | 91.78% | MLP (fold-2) |
| 85.21% | 83.34% | 75.42% | 95.15% | 82.03% | 83.15% | 82.12% | 89.45% | MLP (fold-3) |
| 86.45% | 85.23% | 76.98% | 96.87% | 84.02% | 85.62% | 84.23% | 91.54% | MLP (fold-4) |
| 83.98% | 81.67% | 73.94% | 93.89% | 79.78% | 81.89% | 79.75% | 86.78% | MLP (fold-5) |
| 84.72% | 82.89% | 75.78% | 95.37% | 81.65% | 83.49% | 81.56% | 89.23% | MLP (fold-6) |
| 87.12% | 85.89% | 77.65% | 97.23% | 84.56% | 85.94% | 84.78% | 92.34% | MLP (fold-7) |
| 83.56% | 81.45% | 73.45% | 93.12% | 79.45% | 81.35% | 79.34% | 86.23% | MLP (fold-8) |
| 85.89% | 83.76% | 75.92% | 95.76% | 82.37% | 83.72% | 82.67% | 89.95% | MLP (fold-9) |
| 86.23% | 84.45% | 76.54% | 96.32% | 83.45% | 84.89% | 83.56% | 90.78% | MLP (fold-10) |
| 85.50±1.18% | **83.79±1.26%** | **75.95±1.22%** | **95.91±1.19%** | **82.37±1.25%** | **83.72±1.28%** | **82.65±1.23%** | **89.95±1.22%** | **MLP**  **(Mean ± SD)** |
| 85.52% | 83.72% | 78.95% | 96.32% | 82.84% | 97.49% | 89.32% | 93.41% | Proposed model (fold-1) |
| 87.14% | 85.20% | 81.09% | 98.06% | 84.42% | 99.07% | 90.89% | 95.28% | Proposed model (fold-2) |
| 86.01% | 84.15% | 79.51% | 97.11% | 83.20% | 98.02% | 89.45% | 94.08% | Proposed model (fold-3) |
| 86.99% | 85.08% | 80.87% | 98.03% | 84.38% | 99.01% | 90.76% | 95.07% | Proposed model (fold-4) |
| 85.01% | 83.19% | 78.39% | 96.00% | 82.15% | 97.18% | 88.52% | 92.86% | Proposed model (fold-5) |
| 85.78% | 83.90% | 79.82% | 97.05% | 83.11% | 97.93% | 89.32% | 93.79% | Proposed model (fold-6) |
| 87.32% | 85.47% | 81.52% | 98.41% | 84.62% | 99.28% | 91.23% | 95.52% | Proposed model (fold-7) |
| 84.86% | 82.95% | 78.21% | 95.62% | 81.92% | 96.81% | 88.21% | 92.37% | Proposed model (fold-8) |
| 86.45% | 84.63% | 80.12% | 97.32% | 83.62% | 98.43% | 89.78% | 94.62% | Proposed model (fold-9) |
| 86.81% | 85.32% | 80.69% | 97.85% | 84.14% | 98.92% | 90.34% | 95.12% | Proposed model (fold-10) |
| 86.39±0.85% | **84.52±0.87%** | **79.81±0.84%** | **97.25±0.89%** | **83.62±0.86%** | **98.26±0.88%** | **89.81±0.87%** | **94.32±0.86%** | **Proposed model**  **(Mean ± SD)** |

**Table S4.** Detailed per-class metrices for CHB-MIT dataset.

| **Class** | **Precision** | **Recall** | **F1-Score** | **AUC-ROC** |
| --- | --- | --- | --- | --- |
| **Non-Seizure** | 97.20% | 98.00% | 97.60% | 98.90% |
| **Seizure** | 89.30% | 90.40% | 89.80% | 96.20% |
| **Macro Avg** | **93.30% ± 1.40%** | **94.20% ± 1.30%** | **93.31% ± 1.05%** | **97.55% ± 0.90%** |

**Table S5**. Class-wise Average Performance Metrics on TUSZ Dataset (Mean ± SD, %)

| **Metric** | **ABSZ** | **CPSZ** | **FNSZ** | **GNSZ** | **MYSZ** | **SPSZ** | **TCSZ** | **TNSZ** |
| --- | --- | --- | --- | --- | --- | --- | --- | --- |
| **Matthews Correlation** | 80.53 ± 1.14 | 87.02 ± 0.88 | 85.01 ± 1.06 | 88.21 ± 0.81 | 84.02 ± 1.19 | 86.53 ± 1.03 | 87.51 ± 0.91 | 79.81 ± 1.31 |
| **F1-score** | 78.31 ± 1.29 | 85.07 ± 0.91 | 83.08 ± 1.13 | 86.54 ± 0.73 | 81.52 ± 1.18 | 84.06 ± 1.10 | 85.31 ± 0.82 | 77.53 ± 1.43 |
| **Precision (PPV)** | 74.58 ± 1.22 | 80.58 ± 0.83 | 79.04 ± 0.94 | 82.53 ± 0.72 | 77.01 ± 1.02 | 79.53 ± 0.98 | 81.02 ± 0.75 | 73.04 ± 1.25 |
| **NPV** | 96.03 ± 1.08 | 97.48 ± 0.71 | 96.83 ± 0.91 | 98.01 ± 0.61 | 96.51 ± 1.09 | 97.01 ± 0.85 | 98.32 ± 0.73 | 95.83 ± 1.32 |
| **Sensitivity** | 75.06 ± 1.37 | 83.47 ± 0.89 | 81.54 ± 1.25 | 84.73 ± 0.79 | 79.03 ± 1.32 | 82.03 ± 1.02 | 84.01 ± 0.88 | 74.02 ± 1.49 |
| **Specificity** | 97.02 ± 1.13 | 98.29 ± 0.79 | 97.73 ± 0.86 | 99.01 ± 0.62 | 97.23 ± 1.03 | 98.01 ± 0.83 | 99.01 ± 0.69 | 96.53 ± 1.26 |
| **Accuracy** | 92.51 ± 1.07 | 95.01 ± 0.76 | 93.53 ± 0.91 | 96.02 ± 0.68 | 92.01 ± 1.08 | 94.03 ± 0.91 | 95.21 ± 0.73 | 91.02 ± 1.27 |
| **AUC-ROC** | 93.26 ± 0.94 | 96.04 ± 0.69 | 94.72 ± 0.82 | 96.85 ± 0.61 | 93.88 ± 0.97 | 95.35 ± 0.77 | 96.93 ± 0.66 | 92.24 ± 1.13 |


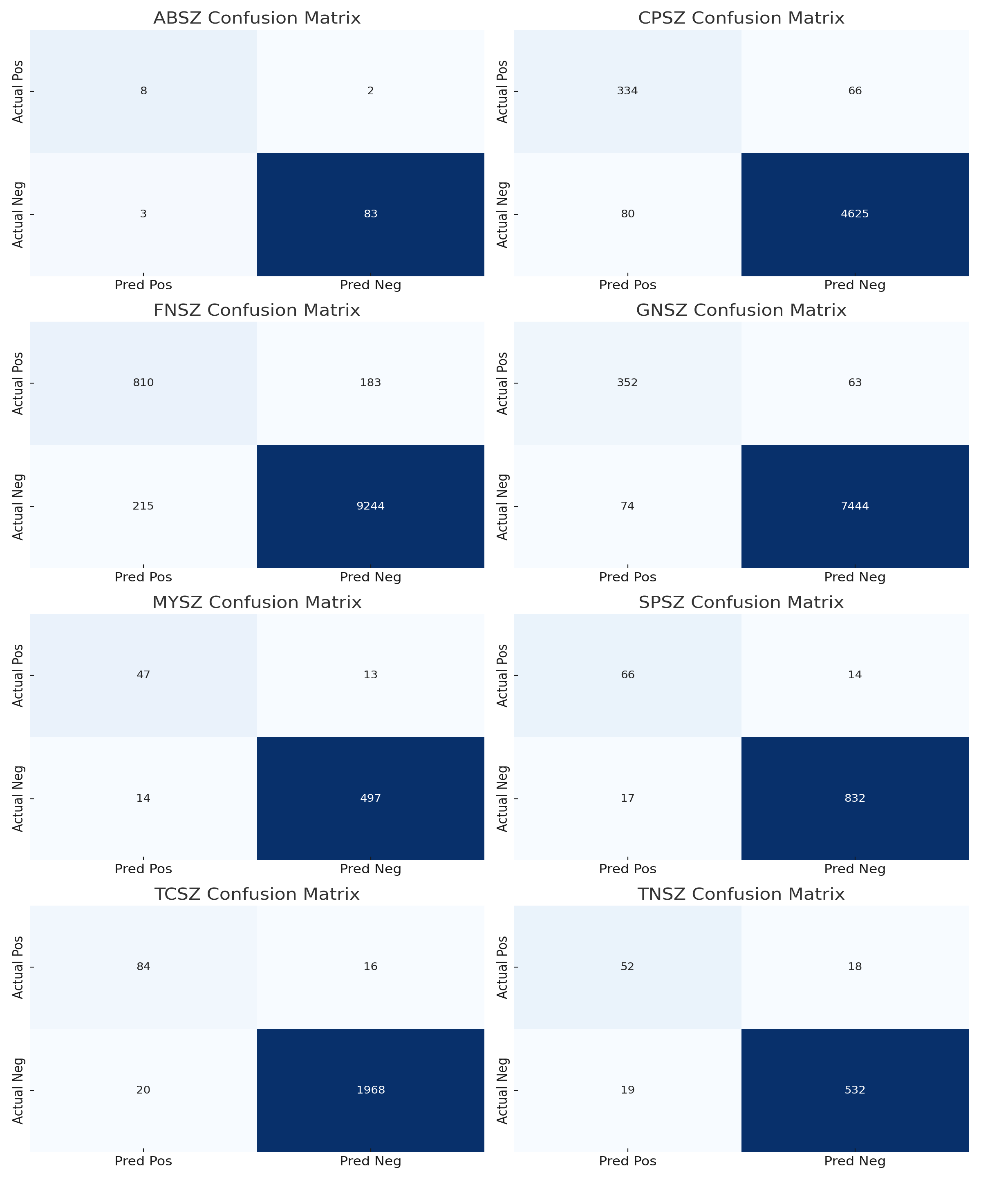


**Fig. S1.** Confusion matrices for different seizure types of TUSZ corpus


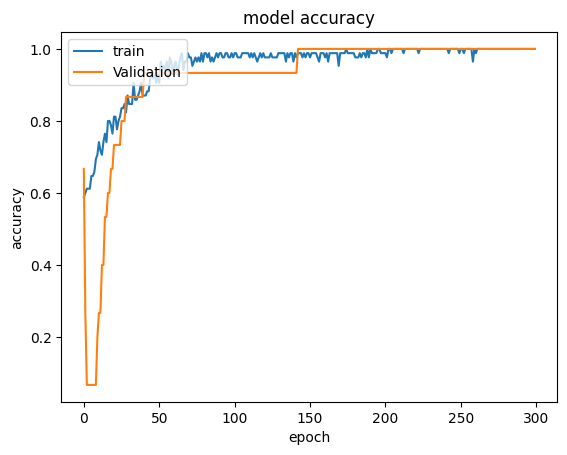

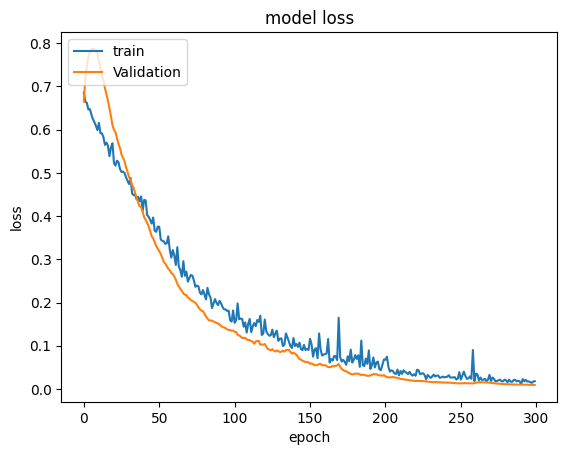


**Fig. S2.** Left: accuracy plot of proposed network on Bonn dataset. Right: loss plot of proposed network on Bonn dataset


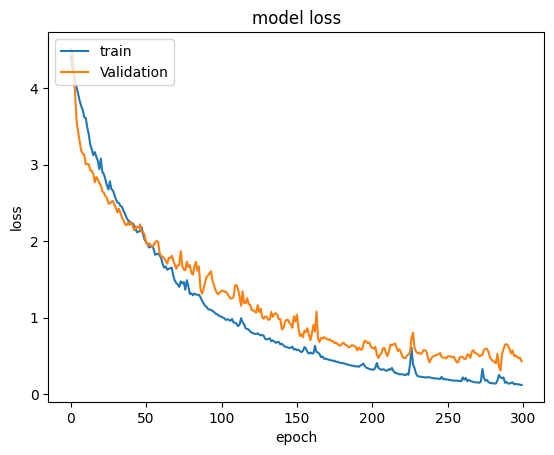

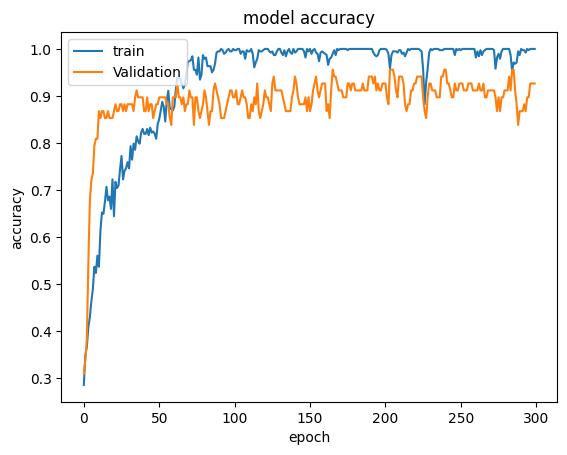


**Fig. S3.** Left: accuracy plot of proposed network on CHB-MIT dataset. Right: loss plot of proposed network on CHB-MIT


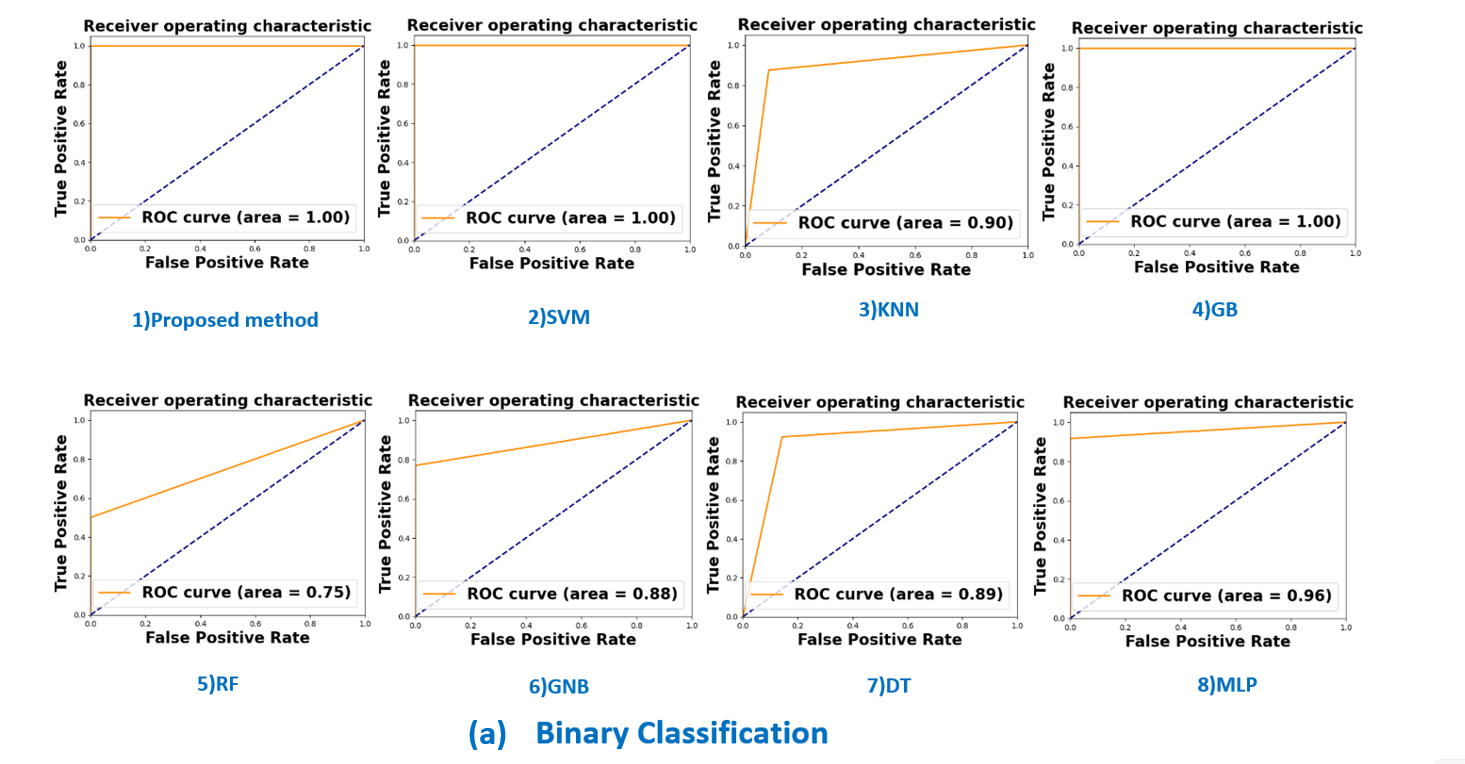


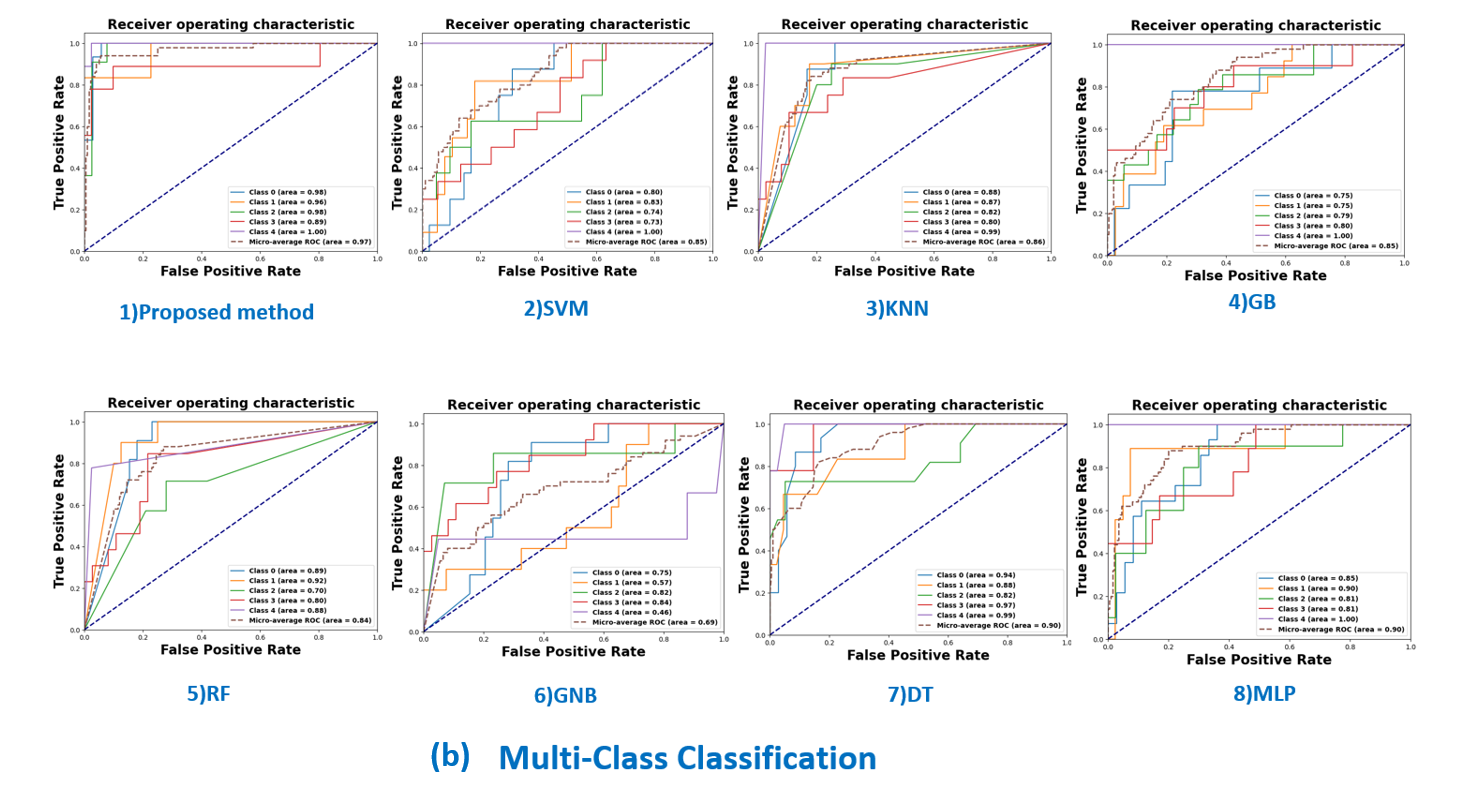


**Fig. S4.** AUC-ROC plots for the proposed model and other machine learning classifiers for Bonn epilepsy dataset (a) Binary Classification, (b) Multi-Class Classification


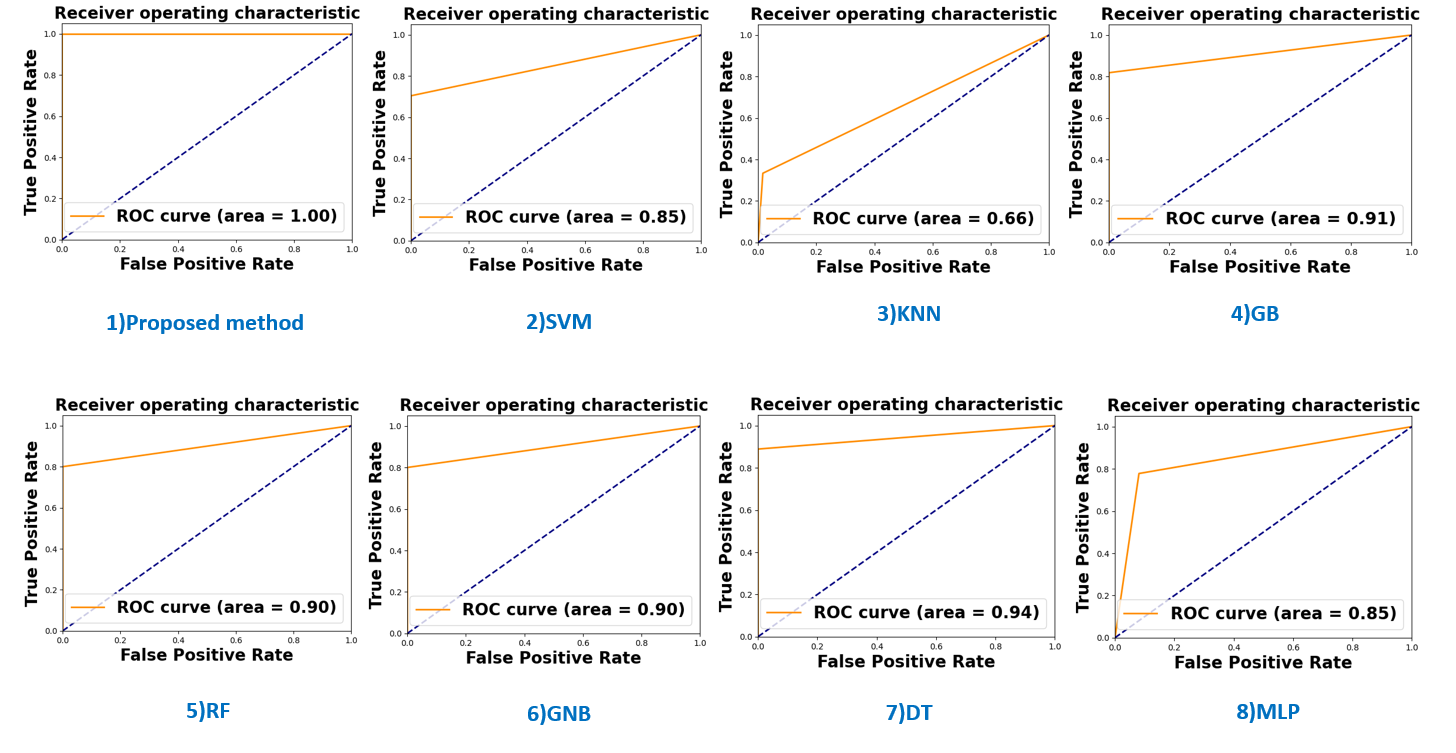


**Fig. S5.** AUC-ROC plots for the proposed model and other machine learning classifiers for CHB-MIT dataset.


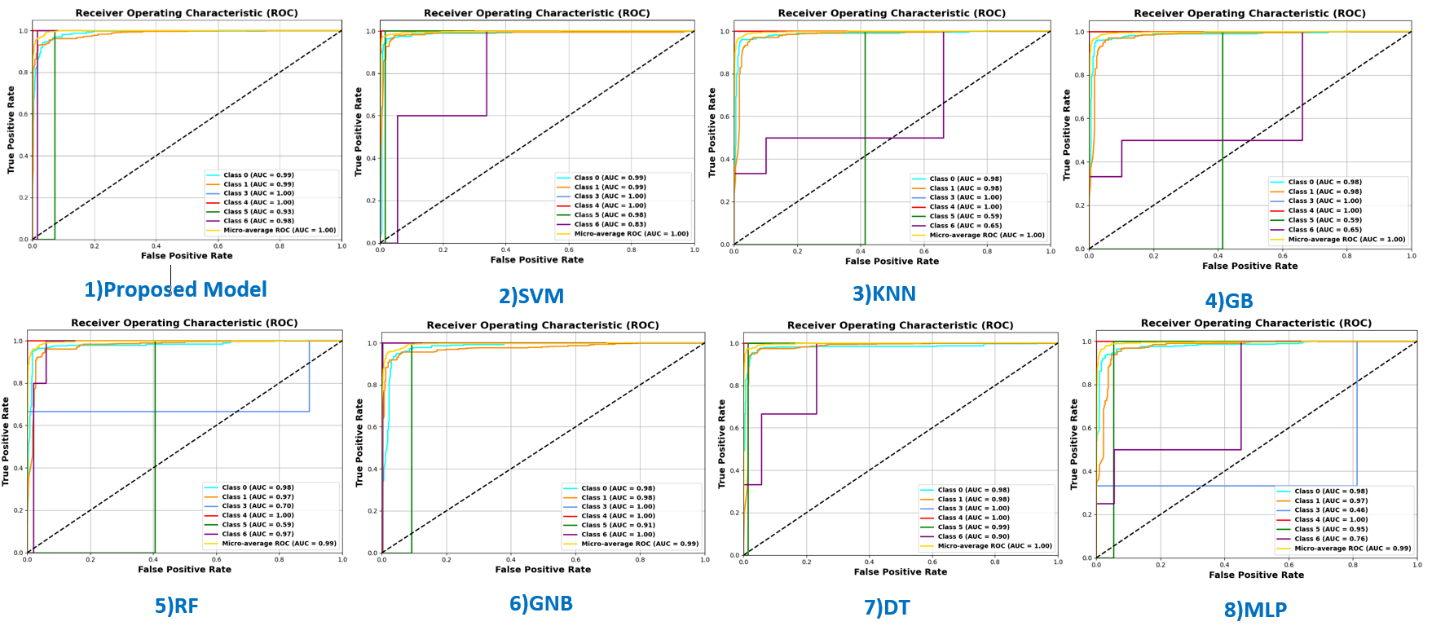


**Fig. S6.** AUC-ROC plots for the proposed model and other machine learning classifiers for TUSZ dataset (multi-class scenario)
